# Testing measurement and structural invariance in latent mediation models – A comparison of IPCR and Bayesian MNLFA

**DOI:** 10.3758/s13428-025-02781-5

**Published:** 2025-08-08

**Authors:** Fabian Felix Muench, Tobias Koch

**Affiliations:** Department of Psychology, Psychological Methods Division, Friedrich-Schiller-Universität Jena, Am Steiger 1, Haus 3, 07743 Jena, Germany

**Keywords:** Latent moderated mediation models, Individual parameter contribution regression, Bayesian moderated nonlinear latent factor analysis, Measurement invariance, Structural invariance

## Abstract

Moderated mediation models are frequently used in psychological research to examine direct, indirect, and total effects across an external moderating variable. When these models involve latent variables, measurement invariance should be tested first to ensure that measures function equivalently across subpopulations. If measurement invariance is violated, conclusions drawn about the moderation effects can be biased. However, measurement invariance is seldom tested across the moderator variable itself, especially if it is continuous. In this paper, we present two approaches that allow testing measurement and structural invariance simultaneously and across continuous covariates. They are termed individual parameter contribution regression (IPCR; Arnold et al., *Structural Equation Modeling: A Multidisciplinary Journal, 27*, 613–628, 2019) and moderated nonlinear latent factor analysis (MNLFA; Bauer & Hussong, *Psychological Methods*, *14*(2), 101–125, 2009). We showcase both approaches with empirical data of $N = 399$ couples in the German Family Panel (Brüderl et al., 2022). We show how MNLFA can be estimated in a Bayesian framework and explain Bayesian model selection with posterior predictive model checks and leave-one-out cross-validation (Vehtari et al., *Statistics and Computing*, *27*(5), 1413–1432, 2017). Afterwards, we present the results of a simulation study comparing IPCR and Bayesian MNLFA with regard to parameter bias. We close with a comparison of both approaches regarding the empirical analysis and the simulation study and provide recommendations for applied researchers working with latent moderated mediation models.

**Supplementary Information:**

The online version contains supplementary material available at 10.3758/s13428-025-02781-5.

Latent mediation analysis and the analysis of indirect effects has a longstanding tradition in the social sciences (Graff & Schmidt, 1982; Jöreskog, 1973) and other disciplines (Wright, 1921). Since its introduction to a psychological audience by Baron and Kenny (1986), it is commonly used to test complex relationships among latent variables (Cheung & Lau, 2008), to evaluate and develop psychological interventions (e.g., Hebbecker et al., 2022), and to investigate potential causal relationships (Imai et al., 2010b; Robins & Greenland, 1992). In this paper, we consider the following prototypical example of a mediation analysis: a personality researcher seeks to examine the relationship between neuroticism (or emotional instability, *X*), fear of love withdrawal (being afraid of losing a romantic partner’s affection, *M*), and partnership autonomy (the extent of feeling independent of one’s romantic partner, *Y*). Figure 1 shows a path diagram of this motivating example, where the variable fear of love withdrawal (*M*) acts as a mediator. Hence, the effect of neuroticism (*X*, exogenous variable) on partnership autonomy (*Y*, endogenous or outcome variable) is mediated through fear of love withdrawal (*M*, mediator). The variables (*X*, *M*, and *Y*) in the model can either be observed or latent. We will assume that the variables are latent and measured using appropriate measurement models. The wording of the items which measure the latent variables is given in Appendix A. Later in the paper, we illustrate this mediation analysis using real-world data from $N = 399$ couples in the German Family Panel (pairfam; Brüederl et al., 2022).

**Fig. 1:**
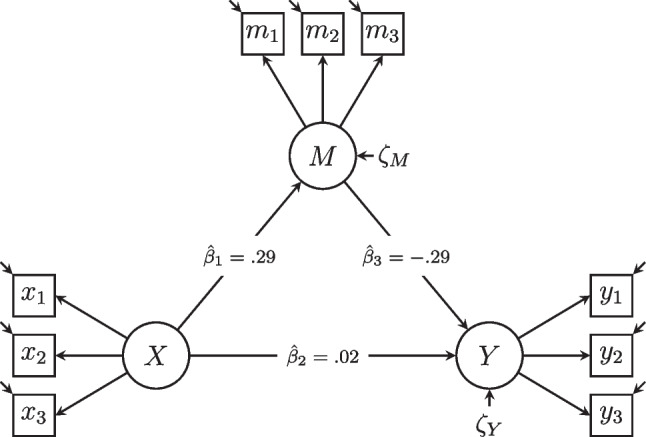
Path diagram of the hypothesized mediation model. Note. *X* = latent neuroticism, *M* = latent fear of love withdrawal, *Y* = latent partnership autonomy. All latent variables are identified by fixing their means to zero and their first factor loading to one. Parameter estimates are unstandardized maximum likelihood estimates

A common and almost natural extension of the above mediation model is a moderated mediation model, which has been studied extensively throughout the last decades (e.g., Feng et al., 2020; Preacher et al., 2007). In a moderated mediation model, one or all direct effect(s) between the latent variables (*X*, *M*, and *Y*) can vary depending on another covariate (moderator, *Z*). As a result, the indirect and total effects of the exogenous variable will also vary based on the values of the moderator. For instance, in a relationship where partners quarrel more frequently (i.e., experience more partnership conflict), greater neuroticism (i.e., emotional instability) might result in increased fear of love withdrawal compared to a relationship with less frequent conflicts. If this is the case, the effect of neuroticism (*X*) on the mediator fear of love withdrawal (*M*) is said to be moderated by partnership conflict frequency (*Z*). The other direct effects in the mediation model may also vary depending on conflict frequency, resulting in indirect and total effects that are moderated as well. Figure 2 illustrates this example in a path diagram.

**Fig. 2:**
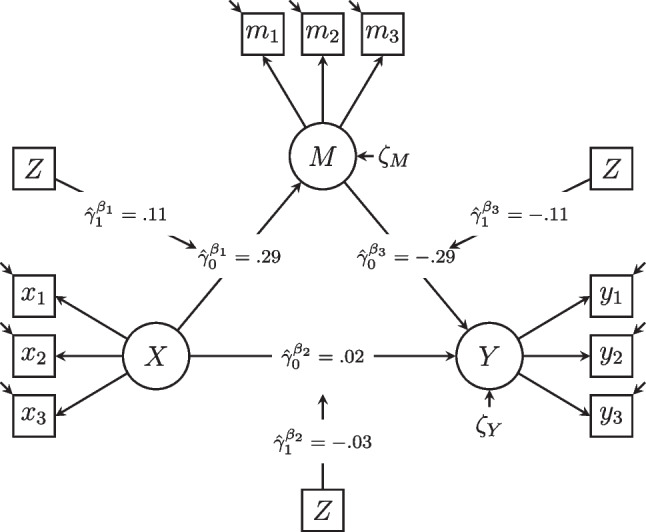
Path diagram of the hypothesized moderated mediation model. Note. *X* = latent neuroticism, *M* = latent fear of love withdrawal, *Y* = latent partnership autonomy, *Z* = manifest partnership conflict frequency. All latent variables are identified by fixing their means to zero and their first factor loading to one. *Z* is grand-mean-centered. Parameter estimates are obtained with individual parameter contribution regression

Moderated mediation analysis has been extended to different measurement designs, for example, multilevel designs (Bauer et al., 2006) or longitudinal designs (Preacher et al., 2010). In addition, moderated mediation analysis has been applied to various research contexts such as personality psychology (e.g., Zhang et al., 2021), clinical psychology (e.g., Zhou et al., 2021), or organizational psychology (e.g., Oh & Roh, 2019). However, the role of measurement invariance in moderated mediation analysis remains relatively unexplored (but see Chen, 2008; Guenole & Brown, 2014; Oberski, 2014; Olivera-Aguilar et al., 2018, for studies regarding either moderation or mediation).

In psychological research, testing measurement invariance (MI) is crucial to ensure that latent variables are measured in the same way across different groups (or time points; Meredith, 1993). MI is often distinguished into four levels (e.g., Meredith, 1993; Widaman & Reise, 1997): (1) configural, (2) weak, (3) strong, and (4) strict MI, and tested subsequently using adequate measurement models. Configural MI is established if a model with the same number of factors and a similar factor loading structure across groups fits the data. Weak MI is achieved if a model with equal factor loadings across groups can be assumed. If this model holds, variances and covariances of the latent variables can be meaningfully compared across groups (Steyer, 1989; Widaman & Reise, 1997). Strong MI is tested using a model that additionally assumes equal item intercepts across groups. If strong MI holds, latent variable means can be meaningfully compared across groups (Widaman & Reise, 1997). Finally, strict MI is established if a model with equal item intercepts, factor loadings, and measurement error variances across groups can be assumed. Although strict invariance has desirable properties, it often fails in empirical applications. After testing for different forms of MI, researchers can proceed to examine differences in structural parameters among latent variables. This analysis is commonly termed structural invariance (SI) testing (Putnick & Bornstein, 2016). For instance, when the primary focus is on regressions among the latent variables (or more specifically, elements of the covariance matrix of latent variables), as it is the case in the latent moderated mediation model, weak MI suffices (e.g., Oberski, 2014). Ignoring MI can lead to bias in structural model parameters, thereby affecting the conclusions researchers draw from their analyses (Guenole & Brown, 2014; Maassen et al., 2023).

We will illustrate the potential problems when ignoring MI testing using an example inspired by Maassen et al. (2023). Consider comparing the latent construct partnership autonomy (the endogenous variable, *Y*) between two (hypothetical) groups of people: those who quarrel with their partner every day, but are quick to reconcile, and those who never quarrel with their partner to avoid conflict. Thus, these two groups are on opposite ends of partnership conflict experiences (the moderator, *Z*). To meaningfully compare the mean and variance of partnership autonomy between these groups, the relationship between the latent construct and its questionnaire items should ideally be the same across groups (i.e., equivalent item intercepts, factor loadings, and variance explained). If we then observe a difference in the distribution of latent autonomy, we can conclude that indeed the construct itself differs between groups, as the relation to its indicators is invariant. Now, imagine that both groups answer to the same item “My partner finds it quite all right if I stand up for my own interests in our partnership”, which is used in the pairfam study to measure partnership autonomy (Brüderl et al., 2022). The first group (who quarrel a lot) would probably answer this item with “absolutely”, as disagreements are part of their daily routine, while the second group would probably answer with “not at all”, as they never quarrel.

However, now imagine that both groups indeed have the same distribution of the latent construct “partnership autonomy” (i.e., experience the same amount of independence in their partnership). In that case, the difference in responses to the autonomy item is due to the different conflict behaviors, and not autonomy. Thus, the item relates differently to the latent construct in both groups, or, in other words, the meaning of the latent variable differs across groups.

In this paper, we compare two recent modeling approaches that allow researchers to simultaneously conduct moderated mediation analysis and test for measurement invariance. The first approach is termed moderated nonlinear factor analysis (MNLFA; Bauer, 2017; Bauer & Hussong, 2009). In MNLFA, all model parameters of a latent variable model can be directly regressed on one or more continuous or categorical covariates. MNLFA is a one-step approach and can be performed using frequentist, e.g., maximum likelihood (ML), or Bayesian, i.e., Markov chain Monte Carlo (MCMC), estimation methods. The second approach is termed individual parameter contribution regression (IPCR; Arnold et al., 2019; 2021) and relies on ML estimation. IPCR is a three-step approach (Arnold et al., 2021), where a hypothesized model (e.g., a mediation model, see Fig. 1) is fit to the data first. Second, individual parameter contributions (IPCs) for each observational unit are approximated from the fitted model. In a third and final step, the IPCs are regressed on one or more covariates. So far, it is not fully clear how IPCR and MNLFA perform when testing measurement invariance as well as moderated mediation hypotheses. Thus, this paper has three main goals: (1) familiarize readers with two important and (relatively) easy-to-use methods for testing measurement and structural invariance, (2) test the performance of both methods in a simulation study, and (3) provide recommendations on when to use which method based on the simulation study.

## Other approaches for exploring parameter heterogeneity

Several other approaches are available to test measurement invariance (MI) or structural moderation hypotheses separately. MI is most commonly tested using a multigroup confirmatory factor analysis approach (Jöreskog, 1971), where independent models are fit for each grouping variable of interest. Model fit between a restricted model, which constrains parameters of the measurement model to equality, and a model that estimates parameters freely is then compared (e.g., by means of a $\chi ^2$-test). A significant decrease in fit of the restricted model indicates a violation of MI (e.g., Meredith, 1993; Widaman & Reise, 1997). Other and newer approaches include multiple indicator multiple causes (MIMIC; Jöreskog & Goldberger, 1975), Bayesian approximate invariance testing (Muthén & Asparouhov, 2012; van de Schoot et al., 2013), multiple group alignment methods (Asparouhov & Muthén, 2014; Marsh et al., 2018), and mixture multigroup factor analysis (De Roover, 2021). Leitgöb et al. (2023) provides an extensive review of both traditional and modern approaches to testing MI.

Moderation of structural parameters, i.e., regressions between latent variables, can also be performed using multiple approaches. The most common include product indicator approaches (e.g., Kenny & Judd, 1984; Marsh et al., 2004) and latent moderated structural equations (Klein & Moosbrugger, 2000). In these methods, regressions between latent variables are moderated by including latent interaction variables in the model. Recently, Rosseel et al. (2025) introduced the structural after measurement approach, a two-stage procedure, which allows for the estimation of a large number of latent interaction effects. A method closely related to the approaches presented here is two-level moderated mediation models with single-level data (Liu et al., 2020, 2022). In this approach, structural model parameters in a mediation model (i.e., the regression coefficients) are allowed to vary across individuals and can be moderated by manifest or latent variables. Liu et al. (2022) shows that the mediation and moderation model can be divided into a two-level structure (with the moderation model at level two), although single-level data are not nested in two levels. This allows to explicitly model heteroscedasticity in the outcome variable and to calculate effect sizes with regard to the moderation effect. Note that in the approaches above, only the regression coefficients in the structural model are moderated, but it is relatively straightforward to include latent moderating variables.

We chose to study MNLFA and IPCR because these approaches enable the moderation of all parameters in a latent variable model across continuous or categorical moderators. Additionally, both approaches allow testing for non-linear moderations (e.g., quadratic, cubic), although we do not demonstrate this here. Technically, MNLFA allows for latent moderators as well, but in practice, we found it hard to moderate all model parameters by a latent variable. To our knowledge, two alternative methods also assess the moderation of all model parameters in latent variable models. The first, local structural equation modeling (LSEM; Hildebrandt et al., 2009, 2016), estimates SEMs sequentially at focal points of a continuous moderator variable, using weighted samples for each focal point (cf. Hildebrandt et al., 2016, pp. 260). For readers interested in detecting MI using LSEM, we refer to related work by Hildebrandt et al. (2009). An R function for fitting LSEMs is available in the sirt-package in R (Robitzsch, 2023b), and a recent article by Robitzsch (2023a) further illustrates LSEM with an empirical example and extensive simulation study. The second collection of methods falls under the term score-based or structural tests (e.g., Zeileis & Hornik, 2007). These methods test for MI or SI across continuous covariates by computing a score function from the case-wise likelihood derivative of an unmoderated factor model. If MI holds, the scores for all observations will fluctuate randomly around zero; if MI is violated, scores will vary systematically across groups (see Merkle & Zeileis, 2013; Wang et al., 2014). An R package for applying score-based tests is available (Zeileis et al., 2002).

## Overview

The remainder of this paper is organized as follows: First, we review the conceptual similarities and differences between MNLFA and IPCR. We explain how either model parameters or individual parameter contributions can vary depending on the values of an external covariate in MNLFA or IPCR, respectively, and how this is related to testing measurement or structural invariance. Second, we illustrate both approaches using real-world data from the pairfam study. To this end, we apply MNLFA and IPCR to our example from the introduction. By incorporating MNLFA in a Bayesian estimation framework, we also demonstrate how the leave-one-out cross-validation information criterion (LOO-IC; Vehtari et al., 2017) can be utilized to determine measurement or structural invariance, serving as a global test of model prediction. Third, we present the results of a Monte Carlo simulation study examining the performance of both approaches under common sample size conditions commonly encountered in psychological research. Finally, we discuss the advantages and disadvantages of both approaches and provide recommendations for applied researchers working with latent moderated mediation models. We also highlight alternative approaches that fall outside the scope of this paper.

## Comparison of IPCR and MNLFA

Both IPCR and MNLFA allow researchers to simultaneously examine measurement and structural invariance, and in particular, moderated mediation hypotheses. Nevertheless, they diverge in their methodologies for achieving this goal. Here, we will discuss the key similarities and differences between the two approaches. To ease the presentation, we first explain how testing moderated mediation hypotheses is similar to testing structural invariance with respect to the latent regression coefficients. We then show how to test measurement invariance assumptions across continuous covariates.

### Testing structural invariance

Referring to our introductory example, the structural regression parameters in a latent mediation model (e.g., Fig. 1) can be linked to continuous or categorical moderator variables using the IPCR and MNLFA approach. For simplicity, we will consider a continuous moderator variable (i.e., partnership conflict frequency, *Z*). Both IPCR and MNLFA allow researchers to include product variables (i.e., interaction terms) between multiple covariates or model nonlinear (e.g., quadratic) relationships between model parameters and covariates. Theoretically, both approaches would also enable researchers to include latent covariates. For an example of including latent covariates within MNLFA using Bayesian estimation, see Oeltjen et al. (2023).

#### Moderated nonlinear latent factor analysis

In MNLFA, the regression coefficients in the latent mediation model can be *directly* related to the moderator variable (*Z*), comparable to the two-level moderated mediation model by Liu et al. (2020, 2022). Here, we only consider linear functions of *Z*, yielding the following equations:

1 $$\begin{aligned} \beta _{1v}&= \gamma _0^{\beta _1} + \gamma _1^{\beta _1} Z_v ~ {(+~\varepsilon _v^{\beta ^1})}, \end{aligned}$$

2 $$\begin{aligned} \beta _{2v}&= \gamma _0^{\beta _2} + \gamma _1^{\beta _2} Z_v ~ {(+~\varepsilon _v^{\beta ^2})}, \end{aligned}$$

3 $$\begin{aligned} \beta _{3v}&= \gamma _0^{\beta _3} + \gamma _1^{\beta _3} Z_v ~ {(+~\varepsilon _v^{\beta ^3})}. \end{aligned}$$

In the above Eqs. 1 to 3, we include an additional index *v* to highlight that the latent regression parameters ($\beta _{1v}$, $\beta _{2v}$, and $\beta _{3v}$) vary depending on the values *v* of the moderator *Z*. In the following, we drop this additional index *v* again, as it is commonly not shown when conducting moderated mediation analysis. As shown in the brackets, it is possible to include additional residual terms in the linear regression equations to account for unexplained parameter heterogeneity in the model parameters. If residual terms are added in the above regression equations, the functions are termed *stochastic* functions, otherwise, they are called *deterministic* functions. Note that the residuals in the above equations also vary across moderator values *v* and not across individuals. This restriction allows us to identify both measurement and structural model parameters for each value $Z_v = z_v$. The MNLFA approach is thus more closely related to a multigroup SEM approach than to the two-level moderated mediation model by Liu et al. (2022).

When moderating the structural regression parameters as in MNLFA (see Eqs. 1 to 3), the resulting model is similar to a moderated mediation analysis, including product variables (or interaction terms; see e.g., Feng et al., 2020). For example, consider the structural model for the mediator variable *M*, which is a linear function of the exogenous variable *X*. Replacing $\beta _1$ (direct effect of *X* on *M*) by $\gamma _0^{\beta _1} + \gamma _1^{\beta _1} Z$ yields a moderation regression, albeit omitting the direct effect of the moderator *Z* on the mediator *M*:

4 $$\begin{aligned} \nonumber M&= \beta _0^M + \beta _1 X + \zeta ^M \\ \nonumber&= \beta _0^M + (\gamma _0^{\beta _1} + \gamma _1^{\beta _1} Z) X + \zeta ^M \\&= \beta _0^M + \gamma _0^{\beta _1} X + \gamma _1^{\beta _1} Z X + \zeta ^M. \end{aligned}$$

In the above equation, $\beta _0^M$ is the intercept, $\gamma _0^{\beta _1}$ is the expected direct effect of *X* on *M* if $Z = 0$, $\gamma _1^{\beta _1}$ is the expected change in the direct effect of *X* on *M* if *Z* increases by one unit, and $\zeta ^M$ is a residual term pertaining to the mediator variable *M*. As such, a moderated mediation analysis can be conceptualized as a particular case of examining structural invariance assumptions regarding latent regression coefficients within a latent mediation model. When the latent regression coefficients vary across the levels of an external moderator (*Z*), it indicates that structural invariance with respect to the latent regression coefficients and the moderator is violated. The direct effects of the mediator *M* (i.e., $\beta _3$) and the exogenous variable *X* (i.e., $\beta _2$) on the endogenous variable *Y* can be linked to the moderator *Z* as well, which yields the following equation:

5 $$\begin{aligned} \nonumber Y&= \beta _0^Y + \beta _2 X + \beta _3 M + \zeta ^Y \\ \nonumber&= \beta _0^Y + (\gamma _0^{\beta _2} + \gamma _1^{\beta _2} Z) X + (\gamma _0^{\beta _3} + \gamma _1^{\beta _3} Z) M + \zeta ^Y \\&= \beta _0^Y + \gamma _0^{\beta _2} X + \gamma _0^{\beta _3} M + \gamma _1^{\beta _2} Z X + \gamma _1^{\beta _3} Z M + \zeta ^Y. \end{aligned}$$

The parameter $\beta _0^Y$ is the intercept, $\gamma _0^{\beta _2}$ is the expected direct effect of *X* on *Y* (if $Z = 0$ and controlling for *M*), $\gamma _0^{\beta _3}$ is the expected direct effect of *M* on *Y* (if $Z = 0$ and controlling for *X*), and $\gamma _1^{\beta _2}$ and $\gamma _1^{\beta _3}$ represent the effects of the moderator variable *Z* on the respective direct effects ($\beta _2$ and $\beta _3$). The residual term of the latent moderated regression analysis is denoted by $\zeta ^Y$. Following a similar logic, other structural parameters in the model (e.g., latent variable means or variances) can also be predicted by *Z*.

#### Individual parameter contribution regression

A major difference between the MNLFA and the IPCR approach is that the latter does not directly link model parameters (e.g., latent regression coefficients) to external variables (see Arnold et al., 2019; 2021). Instead, individual parameter contributions (IPCs) are calculated first based on a model without the moderator. The actual individual model parameters cannot be estimated directly, as there is too little information in the data. In a second step, the estimated IPCs are related to the moderator using conventional ordinary least squares regression analysis. The IPCR approach has been shown to perform similarly to multigroup structural equation models in terms of power and bias (Arnold et al., 2019, 2021). We will now give a brief overview over the calculation of IPCs, as it is done in Arnold et al. (2021). For a more detailed explanation, we refer readers to Arnold et al. (2019).

Let $\ln \mathcal {L}(\varvec{\theta }; \varvec{y}_i)$ be the multivariate normal log-likelihood function. In order to estimate a set of *q* model parameters $\hat{\varvec{\theta }}$, the log-likelihood is maximized. This is done by setting its first-order derivative to zero, i.e.,

6 $$\begin{aligned} \mathcal {S}(\varvec{\theta }) = \left[ \frac{\partial \mathcal {L}(\varvec{\theta }; \mathbf {y_i})}{\partial \theta ^1} \cdots \frac{\partial \mathcal {L}(\varvec{\theta }; \mathbf {y_i})}{\partial \theta ^q} \right] = \varvec{0}, \end{aligned}$$

where $\mathcal {S}(\varvec{\theta })$ is often called the score (e.g., Arnold et al., 2021). In ML estimation, the score function uses data from all individuals $i = 1, \dots , N$ to estimate a single set of model parameters $\hat{\varvec{\theta }}$. In theory, using only data by individual *i* and solving $\mathcal {S}_i(\varvec{\hat{\theta }}) = 0$ would yield individual-specific parameter estimates $\hat{\varvec{\theta }}_i$, but the set of equations is undetermined. However, individual scores $S_i(\hat{\varvec{\theta }})$ can be approximated with a linearization of the score function using the second derivative of the log-likelihood $\varvec{H}(\varvec{\hat{\theta }})$ (the Hessian matrix; for more details, please see Arnold et al., 2021). IPCs can then be calculated using the individual scores and sample Hessian matrix in the following way:

7 $$\begin{aligned} IPC_i(\hat{\varvec{\theta }}) = \hat{\varvec{\theta }} - \left[ \frac{1}{N} \varvec{H}(\hat{\varvec{\theta }}) \right] ^{-1} S_i(\hat{\varvec{\theta }}). \end{aligned}$$

It is worth noting that the mean of the IPCs equals the ML parameter estimates for the whole sample, and the variance of IPCs corresponds to the sandwich estimator of the covariance matrix of parameter estimates (Arnold et al., 2021). Furthermore, as IPCs are calculated based on objective functions (i.e., the ML fitting function or the sum of squared residuals in linear regression models), they are available for all models that can be estimated with ML or ordinary least squares. The calculated IPCs from Eq. 7 can subsequently be regressed on one or more external covariates to examine individual differences.

If there are differences in model parameters across individuals or groups, IPC regression estimates can be biased (Arnold et al., 2019). The authors show that IPC regression estimates deviate consistently from group-specific ML estimates (e.g., in a multigroup SEM), if there are large true individual or group differences in model parameters. This bias increases with the magnitude of differences in model parameters. Arnold et al. (2019) proposed an iterative approach to approximate the IPCs that corrects for this bias by iteratively predicting individual- or group-specific model parameters and re-estimating IPC regression estimates (cf. Arnold et al., 2019).

### Testing measurement invariance

Before testing structural invariance, the researcher should test measurement invariance. Consider the following measurement model for the latent exogenous variable neuroticism (*X*), which is measured by three indicators $x_{i}$:

8 $$\begin{aligned} x_{i} = \alpha _i + \lambda _i X + \varepsilon _{i}, \end{aligned}$$

where the subscript *i* denotes indicators, $\alpha _i$ is the indicator intercept, $\lambda _i$ is the factor loading, and $\varepsilon _{i}$ is a residual with $\varepsilon _{i} \sim \mathcal {N}(0, \sigma _{\varepsilon _{i}}^2)$. For testing MI with MNLFA, all parameters of the measurement model, that is, $\alpha _i$, $\lambda _i$, and residual variation $\sigma _{\varepsilon _{i}}^2$, are again regressed on conflict frequency *Z* in a deterministic (or stochastic) linear fashion:

9 $$\begin{aligned} \alpha _{iv}&= \gamma _0^{\alpha _i} + \gamma _1^{\alpha _i} Z~{(+~\varepsilon _v^{\alpha })}, \end{aligned}$$

10 $$\begin{aligned} \lambda _{iv}&= \gamma _0^{\lambda _i} + \gamma _1^{\lambda _i} Z~{(+~\varepsilon _v^{\lambda })}, \end{aligned}$$

11 $$\begin{aligned} \sigma _{\varepsilon _{iv}}^2&= \exp [\gamma _0^{\sigma _{\varepsilon _i}} + \gamma _1^{\sigma _{\varepsilon _i}} Z~{(+~\varepsilon _v^{\sigma _{\varepsilon _i}})}]. \end{aligned}$$

In the above Eqs. 9 to 11, the parameters $\gamma _0$ are the intercepts and the parameters $\gamma _1$ are the slopes of the moderation regressions (i.e., the effect of the moderator *Z* on measurement model parameters). The subscript *v* indicates again that model parameters vary across values *v* of the moderator. If the moderator *Z* is centered at its grand mean, the intercept $\gamma _0$ can be interpreted as the expected model parameter if *Z* is at the mean level. In the MNLFA approach, an exponential function is used for the moderation of $\sigma _{\varepsilon _{i}}^2$, as variances are naturally bounded by zero. In IPCR, on the other hand, a standard linear function is used, as the IPCs of $\sigma _{\varepsilon _{i}}^2$ rather than the actual model parameters are regressed on *Z*. Note further that in Eqs. 9 to 11, a residual can again be included to allow for unexplained heterogeneity in parameter estimates.

In line with the MI literature, finding no effects of the external covariate *Z* on indicator loadings while allowing the latent variable variance to be moderated freely implies weak MI. Similarly, strong measurement invariance (MI) can be assumed if there is no significant moderation effect on the intercepts while predicting the latent factor mean using the covariate. In the context of MNLFA, it is common not to regress the latent factor on the covariate (i.e., assuming the same factor mean in all groups), but only to predict the intercepts. In this case, strong measurement invariance (MI) may still be present if all intercepts are moderated in exactly the same way so that the latent mean difference in the construct is reflected in the intercepts. Strong measurement invariance (MI) is violated if the effect of the covariate on the intercepts varies across items. Finally, finding no effects on indicator residual variances implies strict MI (Meredith, 1993). MI is usually tested sequentially, starting with configural MI up to strict MI (e.g., Putnick & Bornstein, 2016), although simultaneous procedures are also reported (e.g., Kolbe et al., 2022; Stark et al., 2006). Following the above procedure of moderating the parameters in a latent mediation model, it is possible to test for MI and SI at the same time across one or more continuous covariates using IPCR and/or MNLFA. With the IPCR approach, all IPCs (as approximations of individual or group-specific model parameters) are regressed on the external covariates, as shown above. The moderation regression coefficients can be subjected to *t*-tests to test their respective null hypotheses as well (e.g., $\gamma _1^{\lambda _i} = 0$). MNLFA, on the other hand, allows for a direct (i.e., one-step) moderation of model parameters. It is also possible to moderate only some parameters for which specific hypotheses exist. For MNLFA, we focus on a Bayesian estimation procedure and show how to make use of Bayesian tools for model inspection and evaluation. Furthermore, estimating MNLFA in a Bayesian framework allows for the specification of complex models involving latent moderator variables.^1^ Interested readers can find a recent tutorial on how to test measurement invariance with MNLFA and frequentist ML estimation in Kolbe et al. (2022).

In the following, we will illustrate both approaches with data of $N = 399$ coupled individuals who participated in the pairfam-study (Brüderl et al., 2022). Note, however, that we do not analyze dyadic data here, as it is illustrated by Kenny et al. (2006). All code for the analyses and simulation study is available at https://osf.io/r4e8d/. In the example, we use cross-sectional and observational (non-experimental) data. Hence, we cannot rely on the temporal ordering of variables to determine the direction or causal interpretation of the effects under study. Causal effects are typically defined using potential (or counterfactual) outcomes (e.g., Pearl, 2001; Robins & Greenland, 1992; Rubin, 1974), and causal inferences require strong assumptions (e.g., Muthén et al., 2016; Valeri & VanderWeele, 2013) that are unlikely to be met in our analysis. Unfortunately, a more thorough discussion of this topic is beyond the scope of this article.

## Empirical application with individual parameter contribution regression

IPCR is a fast, simple, and flexible approach to detect model parameter heterogeneity (Arnold et al., 2019). We will now illustrate its use with the mediation example above. To examine measurement invariance, we first specified unidimensional factor models (i.e., for each latent variable separately) in lavaan (Rosseel, 2012). This is suggested by Bauer (2017) for MNLFA, and we also recommend doing so when using IPCR. To identify the latent variables, we set their means to zero and fixed the first factor loading to one. For the remaining measurement model parameters (three item intercepts, two loadings, three residual variances, and the latent factor’s variance), IPCs were calculated first and regressed on the moderator partnership conflict frequency (*Z*) afterwards. The moderator was created by summing individuals’ responses to conflict frequency across five conflict areas and was standardized afterwards. We generally recommend standardizing or centering the moderator for more straightforward interpretation of the moderation regressions’ intercept parameters $\gamma _0$.

For parsimony, we only discuss the moderation coefficients that are important for the MI analysis here. The full results of the IPCR analysis in comparison to MNLFA are given in Table 1. For weak MI to hold, we would expect to find no significant moderation effects of conflict frequency (*Z*) on factor loadings when the factor variances are allowed to vary across moderator values. Differences in scaling of the items could then be expressed in differences in latent variable variances across the moderator. However, if the factor loadings are still significantly moderated, weak MI is violated. Regarding our example, we did not find significant moderations of any of the factor loadings. This means that if the moderator partnership conflict frequency (*Z*) increases by one unit, the factor loadings on the latent variables remain constant across moderator values. Thus, we conclude that weak MI holds for all measurement models across values of conflict frequency (*Z*).

**Table 1:** Parameter estimates of IPCR and MNLFA for the measurement invariance analysis

	IPCR				MNLFA		
Parameter	Est.	*t*	*p*	$95\%$ CI	Post. Mean	$95\%$ CI	Prior
*Moderated factor loadings*							
$\gamma _0^{\lambda _2^X}$	0.82	8.28	0.00	[0.63, 1.02]	0.85	[0.64, 1.09]	$\mathcal {N}(1, .5)$
$\gamma _1^{\lambda _2^X}$	-0.06	-0.60	0.55	[-0.26, 0.14]	-0.10	[-0.33, 0.16]	$\mathcal {N}(0, .5)$
$\sigma ^{\lambda _2^X}$					0.07	[0.00, 0.21]	$\mathcal{H}\mathcal{C}(0, .1)$
$\gamma _0^{\lambda _3^X}$	1.03	8.58	0.00	[0.80, 1.27]	1.11	[0.85, 1.41]	$\mathcal {N}(1, .5)$
$\gamma _1^{\lambda _3^X}$	-0.01	-0.09	0.93	[-0.25, 0.23]	-0.05	[-0.28, 0.25]	$\mathcal {N}(0, .5)$
$\sigma ^{\lambda _3^X}$					0.06	[0.00, 0.18]	$\mathcal{H}\mathcal{C}(0, .1)$
$\gamma _0^{\lambda _2^M}$	0.68	4.79	0.00	[0.40, 0.95]	0.72	[0.46, 1.04]	$\mathcal {N}(1, .5)$
$\gamma _1^{\lambda _2^M}$	-0.08	-0.54	0.59	[-0.35, 0.20]	-0.13	[-0.39, 0.12]	$\mathcal {N}(0, .5)$
$\sigma ^{\lambda _2^M}$					0.18	[0.01, 0.54]	$\mathcal{H}\mathcal{C}(0, .1)$
$\gamma _0^{\lambda _3^M}$	1.06	5.08	0.00	[0.65, 1.47]	1.06	[0.73, 1.48]	$\mathcal {N}(1, .5)$
$\gamma _1^{\lambda _3^M}$	-0.02	-0.08	0.94	[-0.43, 0.39]	0.00	[-0.33, 0.32]	$\mathcal {N}(0, .5)$
$\sigma ^{\lambda _3^M}$					0.22	[0.01, 0.70]	$\mathcal{H}\mathcal{C}(0, .1)$
$\gamma _0^{\lambda _2^Y}$	1.16	7.38	0.00	[0.85, 1.47]	1.18	[0.86, 1.53]	$\mathcal {N}(1, .5)$
$\gamma _1^{\lambda _2^Y}$	-0.15	-0.98	0.33	[-0.46, 0.16]	-0.02	[-0.31, 0.29]	$\mathcal {N}(0, .5)$
$\sigma ^{\lambda _2^Y}$					0.12	[0.00, 0.42]	$\mathcal{H}\mathcal{C}(0, .1)$
$\gamma _0^{\lambda _3^Y}$	1.41	7.23	0.00	[1.03, 1.80]	1.40	[1.04, 1.81]	$\mathcal {N}(1, .5)$
$\gamma _1^{\lambda _3^Y}$	-0.24	-1.24	0.22	[-0.63, 0.14]	-0.08	[-0.44, 0.31]	$\mathcal {N}(0, .5)$
$\sigma ^{\lambda _3^Y}$					0.16	[0.00, 0.52]	$\mathcal{H}\mathcal{C}(0, .1)$
*Moderated indicator intercepts*							
$\gamma _0^{\alpha _1^X}$	2.31	42.39	0.00	[2.20, 2.42]	2.31	[2.20, 2.42]	$\mathcal {N}(3, 2)$
$\gamma _1^{\alpha _1^X}$	0.26	4.81	0.00	[0.16, 0.37]	0.26	[0.15, 0.37]	$\mathcal {N}(0, .5)$
$\sigma ^{\alpha _1^X}$					0.05	[0.00, 0.14]	$\mathcal{H}\mathcal{C}(0, .1)$
$\gamma _0^{\alpha _2^X}$	2.98	53.42	0.00	[2.87, 3.09]	2.98	[2.86, 3.09]	$\mathcal {N}(3, 2)$
$\gamma _1^{\alpha _2^X}$	0.25	4.46	0.00	[0.14, 0.36]	0.25	[0.14, 0.36]	$\mathcal {N}(0, .5)$
$\sigma ^{\alpha _2^X}$					0.05	[0.00, 0.16]	$\mathcal{H}\mathcal{C}(0, .1)$
$\gamma _0^{\alpha _3^X}$	3.33	59.48	0.00	[3.22, 3.44]	3.33	[3.20, 3.45]	$\mathcal {N}(3, 2)$
$\gamma _1^{\alpha _3^X}$	0.25	4.43	0.00	[0.14, 0.36]	0.25	[0.14, 0.36]	$\mathcal {N}(0, .5)$
$\sigma ^{\alpha _3^X}$					0.09	[0.00, 0.22]	$\mathcal{H}\mathcal{C}(0, .1)$
$\gamma _0^{\alpha _1^M}$	1.51	38.18	0.00	[1.43, 1.59]	1.47	[1.39, 1.56]	$\mathcal {N}(3, 2)$
$\gamma _1^{\alpha _1^M}$	0.24	6.00	0.00	[0.16, 0.32]	0.24	[0.15, 0.32]	$\mathcal {N}(0, .5)$
$\sigma ^{\alpha _1^M}$					0.04	[0.00, 0.12]	$\mathcal{H}\mathcal{C}(0, .1)$
$\gamma _0^{\alpha _2^M}$	1.61	35.89	0.00	[1.52, 1.69]	1.59	[1.50, 1.68]	$\mathcal {N}(3, 2)$
$\gamma _1^{\alpha _2^M}$	0.20	4.45	0.00	[0.11, 0.29]	0.19	[0.10, 0.28]	$\mathcal {N}(0, .5)$
$\sigma ^{\alpha _2^M}$					0.04	[0.00, 0.11]	$\mathcal{H}\mathcal{C}(0, .1)$
$\gamma _0^{\alpha _3^M}$	1.56	35.45	0.00	[1.48, 1.65]	1.52	[1.43, 1.62]	$\mathcal {N}(3, 2)$
$\gamma _1^{\alpha _3^M}$	0.17	3.90	0.00	[0.09, 0.26]	0.17	[0.08, 0.26]	$\mathcal {N}(0, .5)$
$\sigma ^{\alpha _3^M}$					0.05	[0.00, 0.15]	$\mathcal{H}\mathcal{C}(0, .1)$
$\gamma _0^{\alpha _1^Y}$	4.16	107.94	0.00	[4.08, 4.24]	4.16	[4.07, 4.25]	$\mathcal {N}(3, 2)$
$\gamma _1^{\alpha _1^Y}$	-0.33	-8.66	0.00	[-0.41, -0.26]	-0.33	[-0.42, -0.24]	$\mathcal {N}(0, .5)$
$\sigma ^{\alpha _1^Y}$					0.07	[0.00, 0.20]	$\mathcal{H}\mathcal{C}(0, .1)$
$\gamma _0^{\alpha _2^Y}$	3.76	83.09	0.00	[3.67, 3.85]	3.76	[3.66, 3.86]	$\mathcal {N}(3, 2)$
$\gamma _1^{\alpha _2^Y}$	-0.32	-7.14	0.00	[-0.41, -0.23]	-0.32	[-0.42, -0.23]	$\mathcal {N}(0, .5)$
$\sigma ^{\alpha _2^Y}$					0.05	[0.00, 0.16]	$\mathcal{H}\mathcal{C}(0, .1)$
$\gamma _0^{\alpha _3^Y}$	4.10	91.70	0.00	[4.01, 4.18]	4.12	[4.02, 4.21]	$\mathcal {N}(3, 2)$
$\gamma _1^{\alpha _3^Y}$	-0.38	-8.57	0.00	[-0.47, -0.30]	-0.38	[-0.48, -0.29]	$\mathcal {N}(0, .5)$
$\sigma ^{\alpha _3^Y}$					0.07	[0.00, 0.18]	$\mathcal{H}\mathcal{C}(0, .1)$
*Moderated residual variances*							
$\gamma _0^{\sigma ^2_{\varepsilon _1^X}}$	0.62	7.23	0.00	[0.45, 0.79]	0.64	[0.48, 0.80]	$\mathcal {N}(0, .5)$
$\gamma _1^{\sigma ^2_{\varepsilon _1^X}}$	0.07	0.82	0.41	[-0.10, 0.24]	0.09	[-0.16, 0.34]	$\mathcal {N}(0, .5)$
$\sigma ^{\sigma ^2_{\varepsilon _1^X}}$					0.08	[0.00, 0.26]	$\mathcal{H}\mathcal{C}(0, .1)$
$\gamma _0^{\sigma ^2_{\varepsilon _2^X}}$	0.87	11.22	0.00	[0.72, 1.02]	0.86	[0.71, 1.03]	$\mathcal {N}(0, .5)$
$\gamma _1^{\sigma ^2_{\varepsilon _2^X}}$	0.02	0.30	0.76	[-0.13, 0.18]	0.01	[-0.16, 0.19]	$\mathcal {N}(0, .5)$
$\sigma ^{\sigma ^2_{\varepsilon _2^X}}$					0.06	[0.00, 0.20]	$\mathcal{H}\mathcal{C}(0, .1)$
$\gamma _0^{\sigma ^2_{\varepsilon _3^X}}$	0.63	7.11	0.00	[0.46, 0.81]	0.60	[0.43, 0.78]	$\mathcal {N}(0, .5)$
$\gamma _1^{\sigma ^2_{\varepsilon _3^X}}$	-0.13	-1.43	0.15	[-0.30, 0.05]	-0.19	[-0.50, 0.08]	$\mathcal {N}(0, .5)$
$\sigma ^{\sigma ^2_{\varepsilon _3^X}}$					0.10	[0.00, 0.37]	$\mathcal{H}\mathcal{C}(0, .1)$
$\gamma _0^{\sigma ^2_{\varepsilon _1^M}}$	0.32	4.42	0.00	[0.18, 0.46]	0.27	[0.17, 0.40]	$\mathcal {N}(0, .5)$
$\gamma _1^{\sigma ^2_{\varepsilon _1^M}}$	0.11	1.51	0.13	[-0.03, 0.25]	0.33	[-0.04, 0.68]	$\mathcal {N}(0, .5)$
$\sigma ^{\sigma ^2_{\varepsilon _1^M}}$					0.32	[0.01, 0.81]	$\mathcal{H}\mathcal{C}(0, .1)$
$\gamma _0^{\sigma ^2_{\varepsilon _2^M}}$	0.67	8.05	0.00	[0.51, 0.83]	0.64	[0.53, 0.76]	$\mathcal {N}(0, .5)$
$\gamma _1^{\sigma ^2_{\varepsilon _2^M}}$	0.17	2.08	0.04	[0.01, 0.34]	0.20	[0.04, 0.37]	$\mathcal {N}(0, .5)$
$\sigma ^{\sigma ^2_{\varepsilon _2^M}}$					0.10	[0.00, 0.33]	$\mathcal{H}\mathcal{C}(0, .1)$
$\gamma _0^{\sigma ^2_{\varepsilon _3^M}}$	0.40	4.90	0.00	[0.24, 0.56]	0.39	[0.27, 0.54]	$\mathcal {N}(0, .5)$
$\gamma _1^{\sigma ^2_{\varepsilon _3^M}}$	0.09	1.07	0.29	[-0.07, 0.25]	0.21	[-0.11, 0.49]	$\mathcal {N}(0, .5)$
$\sigma ^{\sigma ^2_{\varepsilon _3^M}}$					0.27	[0.01, 0.68]	$\mathcal{H}\mathcal{C}(0, .1)$
$\gamma _0^{\sigma ^2_{\varepsilon _1^Y}}$	0.43	7.35	0.00	[0.32, 0.55]	0.41	[0.31, 0.52]	$\mathcal {N}(0, .5)$
$\gamma _1^{\sigma ^2_{\varepsilon _1^Y}}$	0.03	0.55	0.58	[-0.08, 0.15]	0.08	[-0.17, 0.31]	$\mathcal {N}(0, .5)$
$\sigma ^{\sigma ^2_{\varepsilon _1^Y}}$					0.30	[0.02, 0.65]	$\mathcal{H}\mathcal{C}(0, .1)$
$\gamma _0^{\sigma ^2_{\varepsilon _2^Y}}$	0.55	8.25	0.00	[0.42, 0.69]	0.55	[0.44, 0.67]	$\mathcal {N}(0, .5)$
$\gamma _1^{\sigma ^2_{\varepsilon _2^Y}}$	-0.03	-0.43	0.67	[-0.16, 0.10]	-0.05	[-0.29, 0.16]	$\mathcal {N}(0, .5)$
$\sigma ^{\sigma ^2_{\varepsilon _2^Y}}$					0.08	[0.00, 0.25]	$\mathcal{H}\mathcal{C}(0, .1)$
$\gamma _0^{\sigma ^2_{\varepsilon _3^Y}}$	0.40	3.85	0.00	[0.20, 0.60]	0.37	[0.23, 0.54]	$\mathcal {N}(0, .5)$
$\gamma _1^{\sigma ^2_{\varepsilon _3^Y}}$	0.10	0.95	0.34	[-0.11, 0.30]	0.16	[-0.23, 0.49]	$\mathcal {N}(0, .5)$
$\sigma ^{\sigma ^2_{\varepsilon _3^Y}}$					0.55	[0.04, 1.13]	$\mathcal{H}\mathcal{C}(0, .1)$

Note. Est. = IPCR estimate; Post. Mean = posterior mean; $\gamma _0$ = intercepts of moderation regressions; $\gamma _1$ = slopes of moderation regressions; $\sigma$ = unexplained variability in moderated parameters; $\mathcal {N}(\mu , \sigma )$ = normal distribution prior with mean $\mu$ and standard deviation $\sigma$; $\mathcal{H}\mathcal{C}(\mu , \sigma )$ = half-Cauchy distribution prior with mean $\mu$ and standard deviation $\sigma$

For strong MI to hold, we would expect to additionally find no significant moderation effects of conflict frequency (*Z*) on indicator intercepts when the factor means are allowed to vary across the moderator. However, in the unidimensional models fit with lavaan, the factor means are fixed to zero by default and are therefore not moderated by conflict frequency (*Z*) within the IPCR analysis. Hence, strong MI may still hold if all intercepts are moderated in exactly the same way (i.e., same direction and magnitude of the moderation effect). In this case, we can assume that the factor means are linearly moderated, which in turn shows in a moderation of the indicator intercepts, as the factor means are fixed to zero. In our application, this was the case for neuroticism (*X*): we found significant positive moderations of item intercepts, $\hat{\gamma }_1^{\alpha _1^X} =.26$, $t(397) = 4.81$, $p <.001$; $\hat{\gamma }_1^{\alpha _2^X} =.25$, $t(397) = 4.46$, $p <.001$; and $\hat{\gamma }_1^{\alpha _3^X} =.25$, $t(397) = 4.43$, $p <.001$. Indicator intercepts of neuroticism were therefore predicted to be higher the more partnership conflict the individuals experienced. The $95\%$ confidence intervals around parameter estimates (see Table 1) overlapped. Thus, we conclude that strong MI holds across values of conflict frequency (*Z*).

For the mediator fear of love withdrawal (*M*), we also found a significant positive moderation for all item intercepts, $\hat{\gamma }_1^{\alpha _1^M} =.24$, $t(397) = 6.00$, $p <.001$; $\hat{\gamma }_1^{\alpha _2^M} =.20$, $t(397) = 4.45$, $p <.001$; and $\hat{\gamma }_1^{\alpha _3^M} =.17$, $t(397) = 3.90$, $p <.001$. Indicator intercepts of fear of love withdrawal were therefore predicted to be higher the more partnership conflict the individuals experienced. Because the $95\%$ confidence intervals overlapped, we again conclude that strong MI can still be assumed across the values of *Z*. For the endogenous variable partnership autonomy (*Y*), we found significant negative moderations of item intercepts, $\hat{\gamma }_1^{\alpha _1^Y} = -.33$, $t(397) = -8.66$, $p <.001$; $\hat{\gamma }_1^{\alpha _2^Y} = -.32$, $t(397) = -7.14$, $p <.001$; and $\hat{\gamma }_1^{\alpha _3^Y} = -.38$, $t(397) = -8.57$, $p <.001$. Indicator intercepts of autonomy were therefore predicted to be lower the more partnership conflict the individuals experienced. Based on the overlap of the $95\%$ confidence intervals, we conclude that strong measurement invariance (MI) still holds.

For strict MI to hold, we would expect to find no significant effects of conflict frequency (*Z*) on indicator residual variances (in addition to already established weak and strong MI). Indeed, we found no significant moderations of conflict frequency on residual variances of neuroticism (*X*) and partnership autonomy (*Y*). We therefore conclude that strict MI holds. For the mediator fear of love withdrawal (*M*), we found a significant moderation of the second indicator’s residual variance, $\hat{\gamma }_1^{\sigma _{\varepsilon _2}^M} =.17$, $t(397) = 2.08$, $p =.04$, such that the proportion of unexplained variation in this item increased with conflict frequency. We therefore conclude that the measurement model of the mediator is not strictly invariant.

The above tests for parameter invariance indicate that strong MI is supported for all measurement models. The IPCR analyses even support the hypothesis of strict MI for all measurement models of neuroticism and partnership autonomy, but not for the mediator fear of love withdrawal. We will now move on to examine the hypothesized mediation model for moderations on the structural level. To this end, we first specified the latent mediation model in accordance with our theoretical considerations. The hypothesized model fitted the data well, $\chi ^2(24) = 24.22$, $p =.45$, $\textit{CFI} = 1.00$, $\textit{TLI} = 1.00$, $\textit{RMSEA} =.01$, $95\%~\text {CI} = [.00,.04]$. The unstandardized maximum likelihood estimates for the regressions between the latent variables are given in Fig. 1.

In a second step, IPCs were again calculated for this model and regressed on conflict frequency (*Z*). The moderations of the latent regressions $\beta _1$, $\beta _2$, and $\beta _3$ are given in Fig. 2 and in Table 2. Regarding the direct effect $\beta _1$ of neuroticism (*X*) on fear of love withdrawal (*M*), we found a positive (but not significant) moderation by conflict frequency, $\hat{\gamma }_1^{\beta _1} =.11$, $t(397) = 1.91$, $p =.06$. For a mean level of conflict frequency (i.e., when $Z = 0$), the expected direct effect was $\hat{\gamma }_0^{\beta _1} =.29$, $t(397) = 5.04$, $p <.001$, that is, individuals scoring higher on neuroticism had a higher predicted fear of love withdrawal in the relationship. The positive moderation indicates that this effect is predicted to be stronger for individuals with higher conflict frequency. The moderation of $\beta _2$ (the direct effect of neuroticism on partnership autonomy) was small and insignificant. The parameter $\beta _3$ (the direct effect of fear of love withdrawal, *M*, on partnership autonomy, *Y*) was negatively (but not significantly) moderated by conflict frequency, $\hat{\gamma }_1^{\beta _3} = -.11$, $t(397) = -1.30$, $p =.19$. For a mean level of conflict frequency (i.e., when $Z = 0$), the expected direct effect was $\hat{\gamma }_0^{\beta _3} = -.19$, $t(397) = -3.20$, $p <.001$, that is, individuals scoring higher on fear of love withdrawal had a lower predicted partnership autonomy. If conflict frequency increased, the effect of neuroticism on partnership autonomy decreased further (i.e., became more negative). Hence, we would expect individuals who score high on neuroticism and who experience much conflict in their relationship to score high on fear of love withdrawal, and therefore particularly low on partnership autonomy.

**Table 2:** Parameter estimates of IPCR and MNLFA for the structural invariance analysis

	IPCR				MNLFA		
Parameter	Est.	*t*	*p*	$95\%$ CI	Post. Mean	$95\%$ CI	Prior
*Moderated latent regressions*							
$\gamma _0^{\beta _1}$	0.29	5.04	0.00	[0.18, 0.40]	0.24	[0.13, 0.37]	$\mathcal {N}(0, .5)$
$\gamma _1^{\beta _1}$	0.11	1.91	0.06	[0.00, 0.22]	0.09	[-0.02, 0.20]	$\mathcal {N}(0, .5)$
$\sigma ^{\beta _1}$					0.05	[0.00, 0.17]	$\mathcal{H}\mathcal{C}(0, .1)$
$\gamma _0^{\beta _2}$	0.02	0.49	0.62	[-0.07, 0.12]	0.11	[0.01, 0.23]	$\mathcal {N}(0, .5)$
$\gamma _1^{\beta _2}$	-0.03	-0.60	0.55	[-0.13, 0.07]	0.02	[-0.10, 0.13]	$\mathcal {N}(0, .5)$
$\sigma ^{\beta _2}$					0.06	[0.00, 0.18]	$\mathcal{H}\mathcal{C}(0, .1)$
$\gamma _0^{\beta _3}$	-0.29	-3.39	0.00	[-0.46, -0.12]	-0.13	[-0.28, 0.01]	$\mathcal {N}(0, .5)$
$\gamma _1^{\beta _3}$	-0.11	-1.30	0.19	[-0.28, 0.06]	-0.06	[-0.19, 0.07]	$\mathcal {N}(0, .5)$
$\sigma ^{\beta _3}$					0.07	[0.00, 0.23]	$\mathcal{H}\mathcal{C}(0, .1)$
*Moderated latent variances*							
$\gamma _0^{\sigma ^2_{X}}$	0.63	6.40	0.00	[0.43, 0.82]	0.52	[0.36, 0.71]	$\mathcal {N}(0, .5)$
$\gamma _1^{\sigma ^2_{X}}$	0.03	0.28	0.78	[-0.17, 0.22]	-0.01	[-0.40, 0.33]	$\mathcal {N}(0, .5)$
$\sigma ^{\sigma ^2_{X}}$					0.07	[0.00, 0.24]	$\mathcal{H}\mathcal{C}(0, .1)$
$\gamma _0^{\sigma ^2_{M}}$	0.36	3.78	0.00	[0.17, 0.55]	0.28	[0.17, 0.44]	$\mathcal {N}(0, .5)$
$\gamma _1^{\sigma ^2_{M}}$	0.15	1.56	0.12	[-0.04, 0.33]	0.29	[-0.18, 0.69]	$\mathcal {N}(0, .5)$
$\sigma ^{\sigma ^2_{M}}$					0.61	[0.04, 1.27]	$\mathcal{H}\mathcal{C}(0, .1)$
$\gamma _0^{\sigma ^2_{Y}}$	0.27	4.97	0.00	[0.16, 0.38]	0.19	[0.13, 0.27]	$\mathcal {N}(0, .5)$
$\gamma _1^{\sigma ^2_{Y}}$	0.18	3.34	0.00	[0.07, 0.29]	0.35	[-0.03, 0.68]	$\mathcal {N}(0, .5)$
$\sigma ^{\sigma ^2_{Y}}$					0.13	[0.00, 0.48]	$\mathcal{H}\mathcal{C}(0, .1)$

Note. Est. = IPCR estimate; Post. Mean = posterior mean; $\gamma _0$ = intercepts of moderation regressions; $\gamma _1$ = slopes of moderation regressions; $\sigma$ = unexplained variability in moderated parameters; $\mathcal {N}(\mu , \sigma )$ = normal distribution prior with mean $\mu$ and standard deviation $\sigma$; $\mathcal{H}\mathcal{C}(\mu , \sigma )$ = half-Cauchy distribution prior with mean $\mu$ and standard deviation $\sigma$

### Discussion of the IPCR analysis

Taken together, the above IPCR analysis indicates that strong MI is supported for the measurement models of all latent variables (when using a linear moderation regression). We found significant moderations by conflict frequency for all indicator intercepts, but for each respective latent variable, the moderation coefficients showed the same size and magnitude. As the $95\%$-CIs overlap with each other, we retain the null hypothesis of equal moderation coefficients for the respective indicator intercepts. Therefore, we conclude that the moderation is on the level of the latent variable means, and the indicator intercepts are invariant across values of the moderator. Otherwise, we found significant moderations by conflict frequency only for the residual variance of the mediator’s second indicator. Any differences that we found in latent variances, covariances, and latent variable means across values of the moderator were therefore invariant under different scalings of the latent variables (Widaman & Reise, 1997). Hence, we can assume that the direct and indirect effects in the mediation model can be meaningfully compared across values of the moderator and do not reflect measurement differences. Of course, we can never rule out the possibility that there are measurement differences after all, but we do not uncover them in the analysis. For instance, it is possible that there exists a quadratic association between model parameters and the moderator, resulting in a coefficient of zero in a linear regression. Furthermore, we might lack statistical power to detect an effect of the moderator, or failed to test for other relevant covariates.^2^

If full invariance does not hold, researchers can also examine partial measurement invariance (Byrne et al., 1989). A measurement is called partially invariant if only a subset of factor loadings, item intercepts, or residual variances of the respective latent variable significantly differ across groups (or moderator values). In a recent simulation study, Pokropek et al. (2019) could show that even when the majority of indicators were non-invariant, latent variable means and regressions were well-recovered using a partially invariant model (i.e., allowing for these items to be different across groups). However, it is unclear whether it is always justified to treat partial invariance as if full invariance holds, because this issue is highly dependent on the research question at hand (e.g., Widaman & Olivera-Aguilar, 2023). Furthermore, examining partial invariance may be difficult and involves repeated tests of freeing some of the measurement model parameters and keeping others invariant, which can easily produce significant findings by chance (Byrne et al., 1989). To address this problem, the authors recommend employing a cross-validation strategy with a partially invariant model using another independent sample. In fact, IPCR allows for a direct identification of partially invariant latent variables, as all model parameters are simultaneously regressed on the moderator. Nonetheless, the multiple hypothesis tests within IPCR are also susceptible to capitalizing on chance (Arnold et al., 2021). In our example, only the mediator’s second indicator displayed measurement differences across conflict frequency. Using the results from the IPCR analysis, it is also possible to cross-validate this partially invariant model using another data set, although this requires some skill on the user’s side, as there is no option within the ipcr-package.

## Empirical application with Bayesian moderated nonlinear latent factor analysis

MNLFA has been suggested as a general method for testing measurement invariance (Bauer & Hussong, 2009). As in the IPCR analysis, finding significant effects of the moderator on measurement model parameters indicates that MI is likely to be violated. There are recent studies and tutorials on how to implement MNLFA using frequentist (e.g., Kolbe et al., 2022; Kush et al., 2023) and Bayesian (Oeltjen et al., 2023) estimation methods using the R-package OpenMx (Boker et al., 2011) or Mplus (Muthén & Muthén, 2017). In this study, we use Bayesian estimation methods using the Hamiltonian Monte Carlo sampler (Betancourt, 2018) as implemented in the probabilistic programming language Stan (Stan Development Team, 2023b). Bayesian methods offer several advantages, enabling researchers to estimate model parameters even in cases where the maximum likelihood (ML) estimator may encounter difficulties (Lee & Song, 2004). Additionally, a Bayesian framework allows for the inspection of the full probability distributions of model parameters, rather than relying solely on point estimates. In the original MNLFA approach (Bauer & Hussong, 2009), model parameters vary as a deterministic function of the external covariate. Using Bayesian estimation, we can account for additional unexplained parameter heterogeneity by adding a residual to the moderation regressions, as shown in Eqs. 9 to 11. Notably, this residual varies across covariate values, not across individuals.

### Estimation of Bayesian latent factor models

To identify the latent variables and ensure model convergence, we use the same identification conditions as above: the means of the latent variables in the model are fixed to zero, and the first factor loading is fixed to one. Furthermore, in a Bayesian SEM, it is recommended to use informative prior distributions to constrain the factor loadings to only one mode of the posterior distribution due to reflection invariance (e.g., Congdon, 2006; Jackman, 2001; Merkle et al., 2023).^3^ Therefore, the intercepts in the moderation regressions for factor loadings (i.e., the predicted factor loading if the moderator is zero) received normal priors with $\gamma _{0}^{\lambda } \sim \mathcal {N}(1,.5)$ that concentrate most of the probability mass in the positive range of values. To further facilitate estimation, we recommend to use a so-called non-centered parameterization of the model regarding residual parameters (Betancourt, 2020; Papaspiliopoulos et al., 2007). Interested readers may check the Stan model code in the Online Appendix for more details.

In a Bayesian analysis, priors can (and should) be chosen to reflect previous knowledge (Gelman et al., 2017). Specifying these so-called *informative* prior distributions for model parameters can greatly facilitate estimation and increase power (Miočević et al., 2017). If no previous knowledge exists, the current practice is to choose noninformative priors or the software’s default priors (van Erp et al., 2018), although this is usually not recommended in Bayesian SEM (e.g., Miočević et al., 2021; Smid & Winter, 2020). Especially in high-dimensional models, prior choice becomes increasingly important and difficult (Gelman et al., 2017). In the empirical example, we use what may be called *domain-informed* priors (using our domain knowledge; e.g., Gabry et al., 2019). The idea is that instead of using noninformative priors, a prior is constructed that concentrates the probability mass in an area that corresponds to a reasonable data-generating process (Gelman et al., 2017). For example, when using Likert-scale data, the majority of prior probability mass can be concentrated around the range of possible scale values (Smid & Winter, 2020). In our analysis, the weights of the moderation regressions (i.e., $\gamma _1$-parameters) all receive normal priors with $\mathcal {N}(0,.5)$. For scale parameters (i.e., residual standard deviations), we chose half-Cauchy priors (Gelman, 2006) with location 0 and scale .1. The full set of prior distributions for our example is given in Table 1, along with the results of the empirical analysis. If researchers are unsure about their prior choices, we recommend running prior predictive checks (i.e., simulating data from the model using only the prior distributions) and examining whether the predicted values fall within a reasonable range (e.g., Gabry et al., 2019). We provide an example of prior predictive checks for our analysis in the Online Appendix.

### Evaluation of Bayesian latent factor models

Within a Bayesian analysis, posterior predictive model checks (PPMC; e.g., Garnier-Villarreal & Jorgensen, 2020; Gelman et al., 1996; Levy, 2011) can be used to test whether the hypothesized model can reproduce important features of the data, such as means or the general covariance structure. If the model captures the data-generating process well, it should reproduce the features of the data and discrepancies between the model’s predictive distribution and the observed data should only be random fluctuation. PPMCs build on recording a discrepancy measure between the model-implied feature and the data feature at each iteration of the Markov chain. Following Garnier-Villarreal and Jorgensen (2020), the discrepancy measure we focus on here is the difference in model deviance between a saturated model (i.e., no constraints on the mean and covariance structure, $\mathcal {M}_S$) and the hypothesized model $\mathcal {M}_H$, which is the likelihood ratio (*LR*) test statistic in the frequentist framework (see Garnier-Villarreal & Jorgensen, 2020; Levy, 2011):

12 $$\begin{aligned} \Delta _{\textit{dev}} = -2(\mathcal {L}_H - \mathcal {L}_S), \end{aligned}$$

where $\mathcal {L}$ denotes the models’ respective multivariate log-likelihood functions. As the frequentist *LR* statistic, the difference in model deviances will increase with increasing levels of misfit of $\mathcal {M}_H$. In a Bayesian analysis, however, it also has a probability distribution which is centered at zero if $\mathcal {M}_H$ fits perfectly (Hoofs et al., 2018). Therefore, we can analyze the hypothesized model by inspecting the $95\%$ credible interval (CI) of model deviance. If the $95\%$ CI does not include zero, we can conclude that there is some misfit of the model to the data. Furthermore, a posterior predictive *p* value (*PPp* value; Gelman et al., 1996) can be calculated, i.e., from the proportion of Markov chain iterations where the deviance of the hypothesized model was larger than the deviance of the saturated model. A *PPp* value that deviates from the expected 50% if the model’s predictions are in line with the observed data indicates that there is some type of misspecification in the model.

In addition, we use the leave-one-out cross-validation information criterion (LOO-IC; Vehtari et al., 2017) to evaluate and compare competing models. LOO cross-validation assesses the model’s predictive performance by sequentially leaving an observational unit out of the data set, fitting the model to the remainder of the data, and estimating the expected log pointwise predictive density ($\widehat{\text {elpd}}_{\text {loo}}$) for the left-out datum. As this procedure would require re-fitting the model *N* times, Vehtari et al. (2017) proposed to estimate the $\widehat{\text {elpd}}_{\text {loo}}$ for each datum by using pareto-smoothed importance sampling. Summing $\widehat{\text {elpd}}_{\text {loo}}$ across all units yields the expected predictive accuracy for a new data set and can be used for model comparison, similar to the Akaike information criterion (AIC; Akaike, 1973) or deviance information criterion (DIC; Spiegelhalter et al., 2002). A standard error can also be computed for the difference in model $\widehat{\text {elpd}}_{\text {loo}}$ to give a sense of uncertainty of differences in predictive accuracy. We chose to employ LOO-IC for model comparison here because (1) we believe it is the state of the art for assessing a model’s predictive accuracy when using Stan, and (2) the associated loo-package (Vehtari et al., 2017) provides a reliable and easy tool for using the criterion. Following Merkle et al. (2019), we use the marginal log-likelihood for the calculation of the LOO-IC, that is, integrated over latent variables.

### Testing measurement invariance across conflict frequency

As in the IPCR analysis and following Bauer (2017), we started with unidimensional factor models in which all free model parameters were allowed to vary as a function of the moderator conflict frequency (i.e., a configural model). All models were estimated using rstan (Stan Development Team, 2023a) with 6,000 iterations of four Markov chains. Table 1 shows parameter estimates of these models in comparison with the IPCR approach. The parameter estimates are similar to the ones obtained by the IPCR analysis, although 95% CIs are usually wider. This is likely due to the increased model complexity when including residual terms in the moderation regressions. In the simulation study further below, we also examine how excluding these residuals to decrease model complexity affects parameter bias.

For weak MI to hold, we would expect to find no substantial^4^ moderations of factor loadings when the factor variances are also allowed to vary across moderator values. In our example, we found no substantial moderation coefficients (i.e., $\gamma _1^{\lambda }$) for any of the loadings on the latent variables in the model.

For strong MI to hold, we would expect to additionally find no substantial moderations of item intercepts when the factor means are as well allowed to vary across moderator values. As in the IPCR analysis, we can again examine if 95% CIs for the moderation coefficients overlap with each other. We found that the moderation coefficients of conflict frequency (*Z*) on item intercepts of neuroticism (*X*) were different from zero and similar in magnitude and direction, $\gamma _{1}^{\alpha _1^X} =.26$, $95\%~\text {CI} = [.15,.37]$; $\gamma _{1}^{\alpha _2^X} =.25$, $95\%~\text {CI} = [.14,.36]$; $\gamma _{1}^{\alpha _3^X} =.25$, $95\%~\text {CI} = [.14,.36]$. For the mediator fear of love withdrawal (*M*), we also found positive moderations for all item intercepts, $\hat{\gamma }_1^{\alpha _1^M} =.24$, $95\%~\text {CI} = [.15,.32]$; $\hat{\gamma }_1^{\alpha _2^M} =.19$, $95\%~\text {CI} = [.10,.28]$; and $\hat{\gamma }_1^{\alpha _3^M} =.17$, $95\%~\text {CI} = [.08,.26]$, which were all similar in magnitude. For the endogenous variable partnership autonomy (*Y*), we found negative moderations for all item intercepts, $\gamma _{1}^{\alpha _1^Y} = -.33$, $95\%~\text {CI} = [-.42, -.24]$; $\gamma _{1}^{\alpha _2^Y} = -.32$, $95\%~\text {CI} = [-.42, -.23]$; $\gamma _{1}^{\alpha _3^Y} = -.38$, $95\%~\text {CI} = [-.48, -.29]$, which were as well similar in magnitude. Thus, we conclude that strong MI holds for all measurement models.

For strict MI to hold, we would expect to additionally find no moderations of indicator residual variances. Indeed, we found no substantial moderations of conflict frequency (*Z*) on residual variances of neuroticism (*X*). We therefore conclude that strict MI holds across values of the moderator. For partnership autonomy (*Y*), we also did not find moderation coefficients different from zero, although there was substantial variation in residual variances related to *Y*. We will test this finding further below using stepwise tests. Regarding the mediator fear of love withdrawal (*M*), we found a positive moderation of the second indicator’s residual variance, $\hat{\gamma }_1^{\sigma _{\varepsilon _2}^M} =.20$, $95\%~\text {CI} = [.04,.37]$, such that the proportion of unexplained variation in this item also increased with conflict frequency. Hence, we cannot conclude that strict MI holds.

**Table 3:** Model log-likelihoods and $\widehat{\text {elpd}}_{\text {loo}}$ for the MNLFA invariance analysis

Model	Log-Lik	$\Delta _{\text {dev}}$	$95\%$ CI ($\Delta _{\text {dev}}$)	$PPp_{\text {dev}}$	$\widehat{\text {elpd}}_{\text {loo}}$	$\Delta _{\widehat{\text {elpd}}}$	$\widehat{\text {SE}}_{\text {elpd}}$
*Unidimensional factor model, neuroticism (X)*							
Saturated	-1721.53				-1737.62		
Configural MI	-1723.60	4.14	[-19.92, 27.84]	0.63	-1736.75	0.86	1.25
Weak MI	-1722.90	2.72	[-20.57, 25.87]	0.59	-1734.45	2.30	1.00
Strong MI	-1723.25	3.42	[-18.14, 25.53]	0.62	-1732.13	2.32	1.31
Strict MI	-1724.75	6.51	[-15.16, 28.01]	0.73	-1731.14	0.99	2.09
*Unidimensional factor model, fear of love withdrawal (M)*							
Saturated	-1383.97				-1435.62		
Configural MI	-1380.21	-7.52	[-49.93, 35.95]	0.37	-1429.76	5.86	7.33
Weak MI	-1384.32	0.75	[-38.36, 41.24]	0.51	-1427.20	2.56	2.78
Strong MI	-1384.08	-0.40	[-39.48, 38.81]	0.49	-1424.44	2.75	1.74
Strict MI	-1397.39	26.92	[-16.87, 73.46]	0.87	-1434.05	-9.61	6.99
*Unidimensional factor model, partnership autonomy (Y)*							
Saturated	-1408.32				-1453.11		
Configural MI	-1411.73	6.81	[-35.63, 48.99]	0.62	-1455.26	-2.15	3.53
Weak MI	-1412.13	7.06	[-34.87, 51.17]	0.62	-1453.84	1.42	1.12
Strong MI	-1415.91	14.56	[-24.89, 56.25]	0.76	-1453.68	0.16	1.86
Strict MI	-1432.25	47.39	[4.64, 86.91]	0.98	-1449.15	4.53	4.61

Note. Log-Lik = posterior mean of model log-likelihood; $\Delta _{\text {dev}}$ = difference in model deviance to saturated model; $95\%$ CI ($\Delta _{\text {dev}}$) = $95\%$ credible interval for the difference in model deviance; $PPp_{\text {dev}}$ = posterior predictive *p* value for the difference in model deviance; $\widehat{\text {elpd}}_{\text {loo}}$ = expected log pointwise density for leave-one-out cross-validation; $\Delta _{\widehat{\text {elpd}}}$ = difference in $\widehat{\text {elpd}}$ to model before; $\widehat{\text {SE}}_{\text {elpd}}$ = standard error for difference in $\widehat{\text {elpd}}$

#### Stepwise tests

With the MNLFA approach, we can now also employ a step-wise procedure for testing MI before testing for moderations of the structural parameters. The procedure we use here is known as Bayesian *approximate* invariance testing (Muthén & Asparouhov, 2012; van de Schoot et al., 2013), as measurement model parameters are not forced to be strictly equal across values of conflict frequency, but receive a small “wiggle room” (Depaoli, 2021, p. 173). We chose $\mathcal {N}(0,.01)$-priors here, reflecting that moderation effects of .03 or higher on model parameters are thought to be very unlikely. We then carried out posterior predictive checks with regard to the deviance between the hypothesized model and a saturated model (i.e., an unrestricted mean vector and covariance matrix of the manifest variables). Because model parameters in the hypothesized models vary across values of *Z*, the log-likelihood $\mathcal {L}_H$ also varies across values of *Z*, similar to a multigroup SEM. The means and covariance structure in the saturated model $\mathcal {M}_S$ (i.e., a moderated mean vector and covariance matrix) therefore also have to be linearly moderated by *Z* for an adequate comparison. Furthermore, we compared the different MI models in their predictive accuracy using LOO-IC. A higher $\widehat{\text {elpd}}_{\text {loo}}$ indicates a better predictive accuracy of the candidate model, so we retained the model with the highest $\widehat{\text {elpd}}_{\text {loo}}$.

We first tested for configural invariance by specifying unidimensional factor models for each latent variable in which all free model parameters are moderated by conflict frequency in a linear stochastic fashion (i.e., the models shown in Table 1). In our example, the configural models’ deviances for all three latent variables were not substantially different from zero (as indicated by $95\%$ CIs containing zero and posterior predictive *p* values well below the nominal .95; see Table 3), indicating that the general factor structure fitted the data well.

We then tested for weak MI by keeping factor loadings approximately invariant by means of strong priors for the moderation regressions’ slopes $\gamma _1^{\lambda _i}$ and residual standard deviations $\sigma _{\varepsilon }^{\lambda _i}$. Comparing the weak MI models to the saturated models showed that all deviances were still not substantially different from zero (see Table 3). Hence, we conclude that weak invariance also holds for all measurement models.

We tested strong MI by specifying models in which indicator intercepts were now additionally kept approximately invariant. At the same time, we moderated the latent variable means by conflict frequency. Comparing the strong MI models to the saturated models showed that all deviances were still not substantially different from zero (as indicated by $95\%$ CIs and posterior predictive *p* values, see Table 3). We therefore conclude that strong MI holds as well for all measurement models. At last, we tested strict MI by specifying a model in which indicator residual variances were as well kept approximately invariant. Comparing the strict MI models to saturated models showed that model deviances substantially increased for fear of love withdrawal (*M*) and partnership autonomy (*Y*). The posterior predictive *p* value for model deviance of partnership autonomy (*Y*) was .98, indicating that in $98\%$ of Markov chain iterations, model deviance of the strict MI model was larger than for the saturated model. For neuroticism (*X*), deviance was not much larger than in the strong MI model, and the respective posterior predictive *p* value was below the nominal .95. We therefore conclude that strict MI holds for the measures of the exogenous variable. Regarding the large increases in model deviance for the mediator and the endogenous variable, we conclude that strict MI likely does not hold for the respective measures.

By comparing the predictive accuracies with LOO-CV, we found that the strict MI model had the highest predictive accuracy for neuroticism (*X*, $\widehat{\text {elpd}}_{\text {loo}} = -1731.14$, see Table 3). For fear of love withdrawal (*M*), the strong MI models had the highest predictive accuracy, so our conclusion above is also supported by leave-one-out cross-validation. For partnership autonomy (*Y*), the strict MI model had the highest predictive accuracy. However, regarding the substantial increase in model deviance, we retained the strong MI model for this variable.

### Testing structural invariance across conflict frequency

Bayesian MNLFA suggested that strong MI holds for all measurement models. Consequently, we continued to test structural invariance, that is, we now moderated the structural parameters $\beta _1$, $\beta _2$, and $\beta _3$ in a linear stochastic fashion. The results are given in Table 2 along with the results of IPCR. As in the IPCR analysis, we found a positive (but not substantial) moderation of the direct effect of neuroticism (*X*) on partnership conflict (*M*) of the size $\gamma _1^{\beta _1} =.09$, $95\%~\text {CI} = [-.02,.20]$. For a mean level of the moderator (i.e., $Z = 0$), the effect was estimated at $\gamma _0^{\beta _1} =.24$, $95\%~\text {CI} = [.13,.37]$. The positive moderation indicates that this effect is predicted to be stronger for individuals with higher conflict frequency. Furthermore, we found a negative (but not substantial) moderation of the direct effect of fear of love withdrawal (*M*) on partnership autonomy (*Y*), $\gamma _1^{\beta _3} = -.06$, $95\%~\text {CI} = [-.19,.07]$. For a mean level of the moderator, the effect was estimated at $\gamma _0^{\beta _3} = -.13$, $95\%~\text {CI} = [-.28,.01]$. This indicates that for individuals with higher conflict frequency, this effect is predicted to be more negative. The final MNLFA model reproduced the mean and covariance structure well, $\Delta _{\textit{dev}} = 75.13$, $95\%~\text {CI} = [-1.43, 156.42]$. It also had a higher predictive accuracy than a saturated model (in which the mean and covariance structure between the manifest variables were moderated), $\widehat{\text {elpd}}_{\text {loo}}^{\text {final}} = -4601.28$, $\widehat{\text {elpd}}_{\text {loo}}^{\text {sat}} = -4636.04$, $\delta _{\widehat{\text {elpd}}} = -34.76$, $\widehat{SE}_{\text {elpd}} = 13.88$.

### Discussion of the MNLFA approach

Compared to IPCR, MNLFA provides a stepwise approach to testing measurement invariance (MI) and structural invariance (SI), making it more suitable for confirmatory analyses with clear hypotheses about parameter heterogeneity, as demonstrated in our final model. The conclusions from MNLFA align with those of the IPCR analysis: strict MI was supported for the measures of neuroticism (*X*), while strong MI was supported for the measures of fear of love withdrawal (*M*) and partnership autonomy (*Y*). This allows meaningful comparisons of structural regressions between latent variables (i.e., the mediation model) across levels of the moderator (Widaman & Reise, 1997).

The results showed that the direct effect of neuroticism (*X*) on fear of love withdrawal (*M*) was positively moderated by conflict frequency (*Z*): the effect increased with more frequent partnership conflicts and decreased with less frequent conflicts. Similarly, the direct effect of fear of love withdrawal (*M*) on partnership autonomy (*Y*) was negatively moderated by conflict frequency, becoming more negative with frequent conflicts and less pronounced with infrequent conflicts. Since strong invariance was supported, we infer that this moderation occurs at the latent variable level rather than being driven by differences in measurement models.

MNLFA also allows for testing partial MI. For example, to test partial strict invariance (Widaman & Olivera-Aguilar, 2023), we could identify indicator residual variances that remain invariant across conflict frequency levels. The configural models in Table 1 can guide which indicator residual variances to constrain. For fear of love withdrawal (*M*), two residual variances not strongly moderated by conflict frequency could be fixed, while allowing the second residual variance to vary. We could then compare this partially invariant model to a fully invariant one using posterior predictive checks and LOO cross-validation to assess predictive accuracy.

MNLFA is useful for examining parameter heterogeneity, as it allows for testing informed hypotheses. Estimating MNLFA in a Bayesian analysis allows for incorporating prior information, which can be advantageous for estimation and inference. Formulating an informed prior can sometimes be hard, so we encourage researchers to perform prior predictive checks (i.e., sampling from the prior predictive distribution and comparing the samples to actual data Gabry et al., 2019) and posterior predictive checks. A prior sensitivity analysis (e.g., van Erp et al., 2018) is also recommended to check if results change with different informative priors. However, all these steps also render a Bayesian analysis much more intensive than a frequentist one, and may therefore seem daunting for applied researchers. With this contribution, we hope to provide help with a blueprint of an applied analysis and by sharing documented Stan model code in the Online Appendix. In the general discussion, we also recommend a general plan of analysis with both approaches.

## Simulation study

We conducted a Monte Carlo simulation study to compare both the IPCR and the Bayesian MNLFA (BMNLFA) approach in their performance. Previous simulation studies on the IPCR approach (Arnold et al., 2019, 2021) already showed good performance for medium to large sample sizes ($N = 250$ to $N = 1000$). Here, we varied sample size as well, reflecting common sample sizes in psychology, and effect size of one continuous covariate on model parameters. In this article, we only present the most important specifics and results with regard to the simulation study. The full results and discussion are available in the Online Appendix (https://osf.io/r4e8d/).

### Design

We employed a fully crossed simulation design, consisting of the following conditions: (1) the model type, that is, BMNLFA and IPCR, (2) sample size ($N = \{100, 150, 200, 300, 500\}$), and (3) effect size, reflecting a small, medium, or large effect of the continuous covariates on model parameters. For BMNLFA, we also varied if the moderation regressions for model parameters contained a residual term (stochastic variant) or not (deterministic variant). For IPCR, we used both the standard and iterated variant, as proposed by Arnold et al. (2019). This resulted in $2 \times 5 \times 3 \times 2 = 60$ conditions. In a previous simulation, we also used diffuse priors in BMNLFA models for both the deterministic and the stochastic condition, resulting in 30 additional conditions. We also report these results here.

In each condition, we generated $N_{\textit{rep}} = 200$ data sets from a latent mediation in which all model parameters were moderated by one standard normal covariate. Model parameter moderations followed a stochastic process in the data-generating model; that is, a residual term was added to every model parameter moderation regression. Effect sizes were calculated based on unexplained heterogeneity in model parameters and chosen based on the empirical example. We explain the calculation in more detail in the Online Appendix. The population values can be found in Table B1 in Appendix B. In the IPCR conditions, we first fit an unmoderated mediation model to the data using lavaan (Rosseel, 2012). Subsequently, we performed either standard or iterated IPC regression on the resulting model object. In the BMNLFA condition, we fit a fully moderated latent mediation model to the data, allowing for a linear moderation and unexplained heterogeneity in model parameters (a stochastic moderated model). Furthermore, we fit a deterministic model where all model parameters were moderated, but unexplained heterogeneity in model parameters was not allowed. We incorporated the deterministic models in the simulation because they are less parameter-heavy and easier to set up than their stochastic counterparts, thus potentially being more attractive to applied researchers. We assessed performance here based on relative parameter estimation bias (*rpeb*), averaged across parameters of the same type. In the Online Appendix, we also report on 95% coverage rate and power to detect a given effect.

### Results of the simulation study

In the following, we highlight the most important aspects of the results with regard to parameter bias. In general, we only calculated parameter bias if more than 100 of the $N_{\textit{rep}} = 200$ models converged successfully.

#### Convergence

Across all effect sizes and sample sizes, all $N_{\textit{rep}} = 200$ standard IPCR models converged. The iterated IPCR approach, however, displayed major convergence problems with increasing effect size and when the sample size was low. In the large effect size condition, almost none of the iterated IPCR models converged successfully. As iterated IPCR models did not display a convergence rate of more than $50\%$ in all medium and large effect size conditions, we only report results for the small effect size condition here. We discuss this finding further below.

**Fig. 3:**
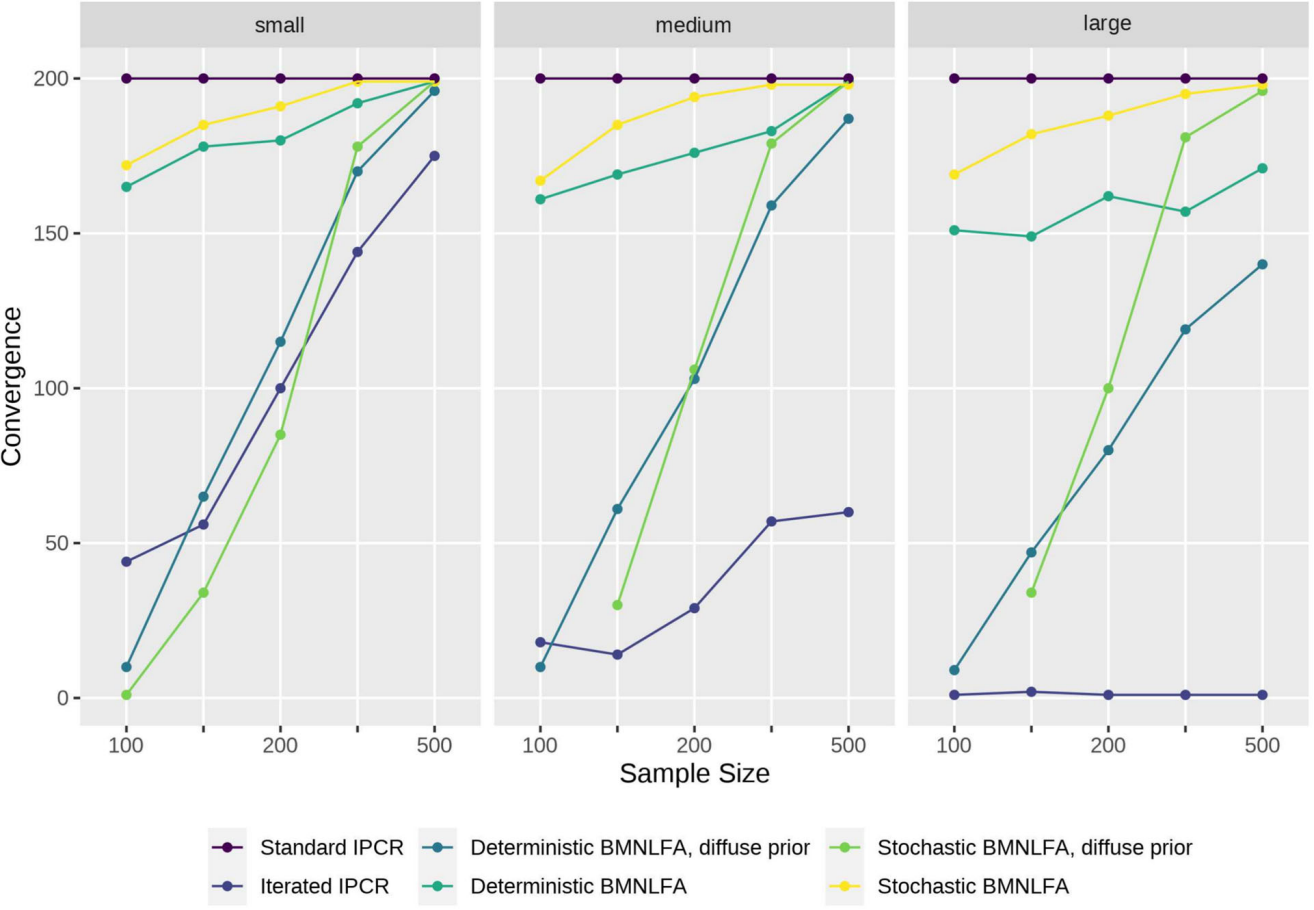
Convergence of all model conditions in the simulation. Note. Sample size has levels $N = \{100, 150, 200, 300, 500\}$

Considering BMNLFA, models with weakly informative priors generally displayed convergence rates above $75\%$. Stochastic models displayed better convergence than deterministic models. Convergence rate was slightly lower for larger effect sizes and increased with increasing sample sizes. In the diffuse prior conditions, almost none of the stochastic models converged in the $N = 100$ sample size condition, but were usually satisfactory starting from $N = 200$. Convergence rates of all conditions are shown in Fig. 3.

#### Bias of measurement model parameters

**Fig. 4:**
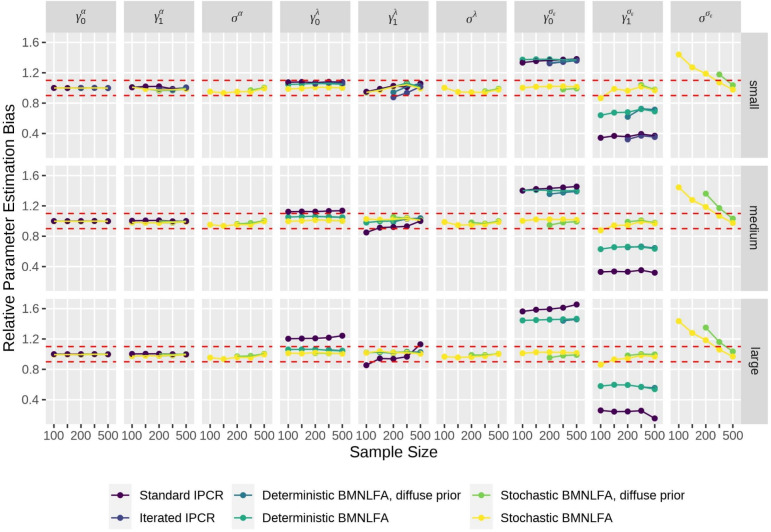
Relative parameter estimation bias of measurement model parameters. Note. $\gamma _0^{\alpha }$, $\gamma _1^{\alpha }$, and $\sigma ^{\alpha }$ = intercept, slope, and residual variation in moderated indicator intercepts. $\gamma _0^{\lambda }$, $\gamma _1^{\lambda }$, and $\sigma ^{\lambda }$ = intercept, slope, and residual variation in moderated factor loadings. $\gamma _0^{\sigma _{\varepsilon }}$, $\gamma _1^{\sigma _{\varepsilon }}$, and $\sigma ^{\sigma _{\varepsilon }}$ = intercept, slope, and residual variation in moderated residual variances. All *rpeb* values are averaged across latent variables. Sample size has levels $N = \{100, 150, 200, 300, 500\}$. The *dashed line* marks an *rpeb* of $10\%$

Across all IPCR and BMNLFA models, moderated indicator intercepts parameters (i.e., $\gamma _0^{\alpha }$, slopes $\gamma _1^{\alpha }$, and $\sigma _{\varepsilon }^{\alpha }$, see first three columns in Fig. 4) usually displayed negligible relative bias across all sample sizes and effect sizes. Bias for moderated factor loadings was small across all IPCR and BMNLFA models when the effect size was small, but increased for IPCR when the effect size increased. For parameters of moderated residual variances, relative bias was small for stochastic BMNLFA models, but consistently above $30\%$ in all IPCR and deterministic BMNLFA models. Columns seven and eight in Fig. 4 show more precisely that while the intercepts $\gamma _0^{\sigma _{\varepsilon }}$ were consistently overestimated, slopes $\gamma _1^{\sigma _{\varepsilon }}$ were consistently underestimated in IPCR and deterministic BMNLFA models. This pattern was more pronounced with increasing effect size.

#### Bias of structural model parameters

**Fig. 5:**
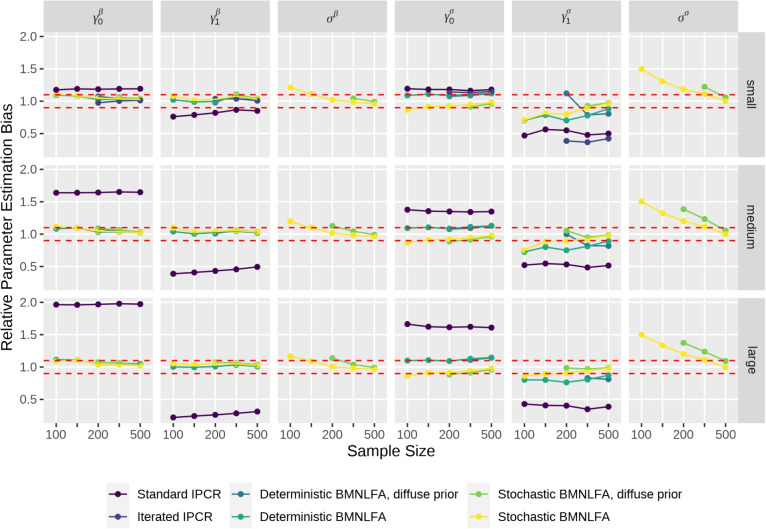
Relative parameter estimation bias of structural model parameters. Note. $\gamma _0^{\beta }$, $\gamma _1^{\beta }$, and $\sigma ^{\beta }$ = intercept, slope, and residual variation in moderated structural regressions. $\gamma _0^{\sigma }$, $\gamma _1^{\sigma }$, and $\sigma ^{\sigma }$ = intercept, slope, and residual variation in moderated latent variable variances. All *rpeb* values are averaged across latent variables. Sample size has levels $N = \{100, 150, 200, 300, 500\}$. The *dashed line* marks an *rpeb* of $10\%$

For parsimony, we averaged relative bias across all moderated structural regressions (i.e., all $\beta$-parameters) and across all moderated latent factor variances (see Fig. 5). In the Online Appendix, we provide separate figures for each moderated structural regression parameter and latent factor variance (i.e., not averaged). Except for standard IPCR models, moderated structural regressions displayed a small relative bias of around $10\%$ for both moderation regression intercepts $\gamma _0^{\beta }$ and slope $\gamma _1^{\beta }$ across all effect sizes. With increasing sample size, bias further decreased. Stochastic BMNLFA models generally displayed small to no bias (see first three columns of Fig. 5). Standard IPCR regression displayed small to medium bias when the effect size of the moderations was small. However, bias increased in IPCR with increasing effect size and followed the same pattern that we also observed for moderated factor loadings and moderated residual variances: while the moderation regressions’ intercepts $\gamma _0^{\beta }$ were consistently overestimated, moderation regressions’ slopes $\gamma _1^{\beta }$ were consistently underestimated. This pattern was also apparent for moderated factor variances. In the IPCR conditions, bias for moderated factor variances was medium to large. In BMNLFA models, bias in moderated factor variances was small starting with a sample size greater than $N = 300$.

### Discussion of the simulation study

We draw the following conclusions from the simulation study: standard IPCR displayed bias with regard to measurement model parameters, which increased with increasing effect size, but decreased with increasing sample size when the effect size was small. Relative bias was most pronounced for moderated factor loadings and moderated residual variances. This might be due to the three-step procedure of IPCR: first, an unmoderated mediation model is fit to the data. On this basis, individual parameter contributions are calculated, which are then regressed on the external covariate *Z* using standard OLS regression. It is therefore likely that bias is already introduced in the first step (i.e., fitting the model with ML estimation in lavaan), which in turn leads to more bias in subsequent IPC regression. This issue is even more pronounced for parameters of the structural model, that is, moderated latent regressions and moderated factor variances. Regarding these parameters, we found that the moderation regressions’ intercepts were overestimated, while the moderation regressions’ slopes were underestimated. This bias increased with increasing effect size and also manifested in low coverage rates (see the Online Appendix). Arnold et al. (2019) developed an iterated IPCR, which can reduce bias in IPCR estimates. In our simulation, the convergence rate for iterated IPCR was satisfactory when the effect size was small with sample sizes greater $N = 200$, but was much smaller for medium or large effect sizes. With large effect sizes on model parameters, almost none of the iterated IPCR models converged. We suspect that having large effects on every model parameter, as in our simulation study, instead of a mix of large and small effects leads to estimation difficulties in the iterated bias-reduction procedure. Arnold et al. (2019) note that the bias in IPC regression estimates increases with the magnitude of differences in individual model parameters. It is possible that the bias-reduction algorithm becomes increasingly unstable as either model complexity or differences in model parameters (or both) increase. However, when the algorithm converged, we could replicate the bias reduction of iterated IPCR, which are reported in Arnold et al. (2019) and Arnold et al. (2021). Furthermore, the IPCR approach is still under active development; hence we expect the iterative algorithm to be more stable in the future.

Regarding BMNLFA, stochastic models (i.e., including a residual in model parameters’ moderation regressions) were generally less biased than deterministic models. This is not surprising, as the data-generating model included unexplained parameter heterogeneity, but the deterministic models did not take this into account. The results with regard to the deterministic BMNLFA models therefore show how well the deterministic approach works if it is wrong in reality. Bias was especially larger in deterministic models for moderated factor loadings and moderated residual variances. Deterministic BMNLFA models can be thought of as a restricted form of stochastic BMNLFA models, with residual variation in model parameters set to zero. If the true data-generating process is a stochastic one, researchers can expect small to medium bias for moderated residual variances and latent variable variances. In stochastic models, which resembled the true data-generating process, bias decreased with increasing sample size. If the focus of the analysis is on weak or strong MI, sample sizes of $N = 300$ seem to be sufficient in terms of parameter bias for stochastic BMNLFA models. Using diffuse prior distributions over model parameters did not introduce more bias, but only threatened model convergence. Especially in stochastic models, using diffuse priors when the sample size was small led to non-convergence of most models.

## General discussion

In the first part of this paper, we presented IPCR and BMNLFA, two approaches for examining parameter heterogeneity across continuous covariates, and used them for analyzing an empirical example. In the second part, we compared their performance in a simulation study. In the following General discussion, we aim to compare both approaches with regard to the empirical application and the results of the simulation study. Furthermore, we recommend a plan of analysis regarding measurement and structural invariance in latent moderated mediation models using both approaches. We also highlight another approach to test for partial invariance which we did not cover in this paper and close with limitations and future directions.

### Comparison of IPCR and BMNLFA

Regarding the empirical application, the results of the IPCR analysis and BMNLFA are relatively similar. With regard to IPCR, we draw the conclusion that strict MI is supported for the measures of neuroticism (*X*) and partnership autonomy (*Y*) across values of conflict frequency (*Z*), and strong MI is supported for the measures of fear of love withdrawal (*M*). With regard to MNLFA, we draw the conclusion that strict MI is supported for the measures of neuroticism, and strong MI is supported for the measures of fear of love withdrawal and partnership autonomy. Subsequently, the moderations of structural parameters (i.e., regressions or factor variances) were examined. IPCR and BMNLFA also seem to agree here: the regression coefficient $\beta _1$ (neuroticism, *X*, to fear of love withdrawal, *M*) was positively moderated by conflict frequency (*Z*), such that the effect is more pronounced for individuals displaying more conflict frequency. The coefficient $\beta _3$ (fear of love withdrawal, *M*, to partnership autonomy, *Y*) was negatively moderated by conflict frequency, such that the effect was as well more pronounced (i.e., larger in magnitude) for individuals displaying more conflict frequency. However, this is only descriptive evidence, as $95\%$ CIs included zero for all moderation effects.

The results of the simulation study suggest that especially structural model parameters display bias in standard IPCR for medium to large effect sizes. It seems that the moderation regressions’ intercepts $\gamma _0$ are overestimated in the first step (i.e., within lavaan) and the slopes $\gamma _1$ are subsequently underestimated by IPCR. This bias in estimation gets more severe when the effect of the external moderator *Z* increases, as exemplified in Fig. 5. However, bias in model parameters is usually comparable to bias in BMNLFA when the effect size is small, which was the case in the empirical application. BMNLFA models generally displayed less bias than IPCR, with stochastic models performing best in all scenarios. This is not surprising, as a stochastic moderated mediation model was the true data-generating model in the simulation study. We included a deterministic BMNLFA condition in the simulation to test how large the parameter bias would be in comparison to stochastic BMNLFA models. Including an error term in each model parameter’s moderation regression is computationally demanding and tends to lead to increased posterior *SD*s, which in turn makes it harder to detect a given effect to be substantially different from zero. When a deterministic model is used instead of a stochastic one (and the data-generating process is stochastic in nature), bias increased mostly for moderated residual or latent variances. Thus, when the focus is on testing only weak or strong MI, it can also be justifiable to drop the residual term and fit a deterministic moderation regression to reduce model complexity.

Ignoring MI may lead to bias of different magnitudes in structural model parameters, ranging from acceptable to severe (Guenole & Brown, 2014). The authors suggest that modeling MI if it is violated is the safest way to deal with it. Hence, in the final model (see Table 2), we included conflict frequency (*Z*) as a moderator for the residual variances of fear of love withdrawal (*M*) and partnership autonomy (*Y*). Latent neuroticism (*X*) appeared to satisfy strict MI in our analyses, so we fixed the moderator’s effect on measurement model parameters to zero.

As we have shown, the inclusion of a continuous variable to moderate model parameters is possible with both IPCR and MNLFA. However, both approaches differ in two important aspects: first, in their fundamental type of analysis, and second, in the manner in which moderation parameters are estimated. IPCR is at heart an exploratory procedure (Arnold et al., 2021). As such, it is suited to explore the effect of several covariates of interest on model parameters and derive data-driven hypotheses. The estimation of IPC regression proceeds in three steps: first, an unmoderated hypothesized model is fit to the data. For this model, individual parameters are approximated and in a third step regressed on one (or multiple) external covariates (Arnold et al., 2019). In the ipcr-package (Arnold et al., 2019), all model parameters are moderated by default. This has the advantage that the moderator is automatically controlled for in both the measurement and in the structural model. Furthermore, IPCR is a very fast and flexible procedure with an easy-to-use package interface. The ipcr-package is compatible with all linear models fitted with the standard lm()- or glm()-functionality in R and with all structural equation models fitted with lavaan and can therefore handle a large proportion of models fitted by applied psychological researchers.

However, there are some limitations to testing MI with the IPCR approach. First, MI cannot be tested in a stepwise procedure, as it is usually done, because all model parameters are regressed simultaneously on the external covariate. Second, and related to the first point, there is no global model test available to compare the fully moderated model to a simpler one, where only a few parameters are moderated. Theoretically, the predictive accuracy of a moderated SEM that is analyzed with IPCR could be examined with leave-one-out cross-validation. However, for SEMs fitted with frequentist estimators, cross-validation is seldom used for model comparison, especially when the sample size is small. Furthermore, there is also no easy implementation to allow for this endeavor. Third, as a data-driven procedure, IPCR may also be susceptible to capitalizing on chance and overfitting to random fluctuations in the data (see Arnold et al., 2021, p. 381). To avoid this, the authors propose a regularized IPC regression that penalizes model complexity. Fourth, Arnold et al. (2019, p. 619) show that IPCR parameter estimates are not guaranteed to reflect the true individual parameter values and can hence be inconsistent. This inconsistency can be tackled by using iterated IPCR, as shown in Arnold et al. (2019) and Arnold et al. (2021). However, iterated IPCR displayed convergence problems in our simulation when effects on moderated model parameters increased. At last, the multiple testing procedure might result in type I errors (Arnold et al., 2019). However, it could be shown in a simulation study that type I error rates were always close to the conventional 5% (Arnold et al., 2021).

MNLFA, on the other hand, is primarily a confirmatory approach. Model parameters are also estimated in one step, where the moderation regressions are directly included within the latent mediation model. This has the advantage that the user can compare competing models where only specific model parameters are moderated via model deviance or leave-one-out cross-validation. LOO-CV can be used in a Bayesian analysis to select between competing models by comparing their predictive accuracies and has been shown to perform well for comparing SEMs (Lu et al., 2017). The recently introduced approximation of exact LOO-CV by pareto-smoothed importance sampling avoids re-fitting the model for each left out datum and is easily accessible by applied researchers with the loo-package (Vehtari et al., 2017). Furthermore, Bayesian MNLFA allows explicitly modeling unexplained parameter heterogeneity in the form of a residual in moderation regressions (note, however, that it is possible to extract an $R^2$ of the linear regressions from IPCR models as well). In Bayesian MNLFA, it is also possible to include a latent moderator variable, which is, to the best of our knowledge, not possible with either IPCR or MNLFA relying on ML estimation. We provide code for an example using a latent moderator in the Online Appendix. However, there is not yet an R-package that allows for an easy setup of BMNLFA (but note the mnlfa-package by Robitzsch, 2024, for moderated item response theory models with ML estimation). Especially when an error term is included in moderation regressions of stochastic models, BMNLFA becomes quite parameter-heavy and can be difficult to estimate for the MCMC sampler. This is reflected in low model convergence rates when using only diffuse priors in the simulation study.

The choice of adequate prior distributions itself can prove a difficult task and should be accompanied by prior predictive checks or prior sensitivity analyses, as shown for example by van Erp et al. (2018). We provide code for prior predictive checks for our example in the Online Appendix. At last, while Bayesian latent factor models gained popularity across the last decade in psychology, they still remain relatively understudied and underused in comparison to their frequentist counterparts. Levy (2011) and Garnier-Villarreal and Jorgensen (2020) are two important contributions that derive popular frequentist model fit indices in a Bayesian context. Most of them are by now included in the blavaan-package (Merkle & Rosseel, 2018). However, blavaan does not allow fitting moderated factor models yet. In the case of BMNLFA, including a deviance test requires fitting a moderated saturated model, in addition to the moderated hypothesized model, which is a non-trivial endeavor and can significantly increase model run time. In the Online Appendix, we include all Stan model code of both the empirical application and the simulation study, hoping to facilitate this task for applied researchers.

#### Recommendations for applied researchers

With this contribution, we by no means want to give the impression that IPCR and BMNLFA are two polar choices of which researchers have to choose one in particular for their analyses. However, we believe that the flexibility, speed, and ease of use render IPCR especially useful for an exploratory analysis. This is in line with the recommendations given by Arnold et al. (2021). For instance, if a latent mediation model representing a given scientific theory is being tested by means of structural equation modeling, the effect of additional covariates that were not part of the original hypotheses can be explored with IPCR. As such, these analyses could help develop further research questions. Of course, the IPCR analyses should then be labeled as exploratory (Arnold et al., 2021). Note, however, that based on the simulation study, we recommend sample sizes larger than $N = 500$ to reliably detect given effects with a power greater than .8 (see the simulation study in the Online Appendix).

MNLFA, on the other hand, is more often used as a confirmatory tool. As discussed above, MNLFA allows for a step-wise test of competing models, which is the usual way of testing MI, by means of model deviance or the LOO-IC. Thus, researchers can test different forms of MI, inspect parameter posterior distributions, and include only substantial moderations in the final model in which hypotheses regarding structural moderations are tested. Ultimately, MNLFA allows for a more precise test of hypotheses regarding moderations of only specific parameters (i.e., structural regression coefficients) than IPCR. To test the model’s fit to the data, posterior predictive checks can be conducted by comparing model deviance to that of a saturated model. We believe this to be an informed analysis procedure, which is in line with recent recommendations for Bayesian data analysis (e.g., Gelman et al., 2020). Furthermore, priors can be chosen that reflect a certain degree of belief about the model parameters (and hence, the data generating process). In moderated latent variable models, we encourage researchers to choose priors that correspond to a reasonable range of values with regard to the scales used in the analysis. For example, if a five-point Likert scale ranging from 1 to 5 is used, the bulk of predicted values from the prior predictive distribution should be within this range (e.g., Smid & Winter, 2020).^5^ Gabry et al. (2019) provides an excellent tutorial on a workflow in a Bayesian analysis, including prior and posterior predictive checks (albeit with different data than usually obtained by psychological researchers).

As of now, there is no clear consensus on what steps to follow when measurements are non-invariant. Testing for partial invariance, that is, holding only some of the measurement model parameters equal across groups, is a step that is often taken after measurements are found to be non-invariant. However, even in their initial contribution on this topic, Byrne et al. (1989) warned that post-hoc analyses of partial invariance are in danger of capitalizing on chance, especially if many competing models are tested. They argue for employing a cross-validation strategy, where a data set is split in two and the first sample is used for exploratory analyses. The second sample can then be used to test the hypotheses generated from the exploratory analyses in a confirmatory fashion. In fact, we believe that this procedure can also fit well in an analysis employing both IPCR and MNLFA. Exploratory research questions regarding the moderation of measurement and structural parameters can be tested using IPCR with the first dataset, as it is quick and easy to use. With the second data set, informed hypotheses can then be tested with a reasonable MNLFA model. Estimates from the IPCR analysis may even be used to construct informed prior distributions for the MNLFA model, as in the Empirical Bayes method (see, e.g., van Erp et al., 2018, for empirical Bayes priors specifically related to Bayesian SEM).

To answer the problems related to partial invariance testing, Asparouhov and Muthén (2014) developed multiple group factor analysis alignment, which was predominantly an exploratory procedure at first. It allows for the estimation and comparison of group-specific factor means and variances when strong measurement invariance is not supported by the data. The alignment method makes use of a loss function to choose factor means and variances in such a way that the amount of non-invariance in measurement model parameters is kept to a minimum (cf. Asparouhov & Muthén, 2014, p. 3). This results in measurement model parameters that are “aligned” across groups (hence the name; Asparouhov & Muthén, 2014). Marsh et al. (2018) extended the approach to a confirmatory procedure and tested it in a large-scale study of international student achievement. In a simulation study, they also demonstrated that it is less biased concerning factor means than partial invariance models. Factor analysis alignment is a very flexible and promising procedure and can handle a large number of groups. However, to the best of our knowledge, it has only been applied and tested in relatively large studies (starting with $N = 1500$; Marsh et al., 2018). The properties of alignment in small-sample studies are yet to be systematically examined. Unfortunately, including the alignment method in our present simulation study for small-sample performance was beyond the scope of this article.

### Limitations and future directions

There are some limitations to this contribution that we would like to address. First, we cannot provide the data we used in the empirical application, as we do not own it. However, we provide a data set in the Online Appendix which we generated from the MNLFA analysis final model’s posterior predictive distribution. Second, we only investigated the effect of one moderating variable in the empirical example. It is likely that the relationships between the latent variables are more complex than we assumed in the example, and there may be more moderation or mediation effects. Hence, the results of our analysis are only valid for the specific model structure we investigated. Third, and related to the second point, we cannot draw any causal conclusions from the analysis. As we noted above, there is a distinction between merely predictive and causal effects. In order to interpret the effects of our analyses as causal, important assumptions have to hold, some of which cannot be tested empirically (Mayer et al., 2014; Muthén et al., 2016). Uncovering an effect that is really causal is notoriously hard, and social scientists may even avoid causal language entirely (Rohrer, 2018). We agree with Rohrer (2018) that researchers can considerably improve their analyses when explicating the causality assumptions involved. Furthermore, researchers can also include sensitivity analyses (Imai et al., 2010a) to increase confidence in causal associations. Unfortunately, both are beyond the scope of this article. Ultimately, we did not investigate the performance of the LOO information criterion in the simulation study presented here. Its use in latent factor models in psychology has only recently begun and remains understudied. Future simulation studies are needed to investigate the performance of the LOO-IC concerning model selection in psychological research.

### Conclusion

In this paper, we presented IPCR and BMNLFA, two approaches for examining parameter heterogeneity across continuous covariates. By showing their application using empirical data, we want to aid researchers to make use of one or both approaches when faced with testing MI across continuous covariates instead of categorizing them and losing precious information. With the simulation study, we want to give researchers an impression of what bias to expect under different data conditions. All code, figures and tables that are not already presented here can be found in the Online Appendix. With these materials, we hope to help with the use of both approaches.

## Supplementary Information

Below is the link to the electronic supplementary material.

Supplementary file 1 (pdf 1331 KB)

## Data Availability

The data set analyzed in this study is not publicly available due to copyright reasons. However, we created a synthetic data set that allows readers to check the correctness of their implementation. The data set is available in the Online Appendix (OSF repository; https://osf.io/r4e8d/).

## Footnotes

1 We provide code for an MNLFA model with a latent moderator in the Online Appendix, but do not demonstrate it here.

2 We thank an anonymous reviewer for pointing these out.

3 Reflection invariance describes the problem that latent factors and factor loadings can only be identified up to an orthogonal rotation (Erosheva & Curtis, 2017; Jackman, 2001). This may lead to non-convergence of the Markov chain, as there is more than one set of parameters that produces the same joint model likelihood.

4 We use the term “substantial” here to refer to 95% CIs not containing 0, similar to “significant” in the frequentist framework.

5 Note, however, that if a multivariate normal model is used, predictions for item responses will naturally extend the scale limits because of the distribution’s infinite support. To predict integer item responses, polytomous item response theory models like the graded response model can be used.

## Code Availability

The code to reproduce the analyses in this study is available in the Online Appendix (OSF repository; https://osf.io/r4e8d/).
