# Supplementary material for: Testing measurement and structural invariance in latent mediation models – A comparison of IPCR and Bayesian MNLFA
Source: Behav Res Methods. 2025 Aug 8;57(9):250. doi: 10.3758/s13428-025-02781-5 (PMC12334378; doi:10.3758/s13428-025-02781-5)
Supplement: Supplementary file 1 — (pdf 1331 KB) [file 13428_2025_2781_MOESM1_ESM.pdf]

Appendix A

Items

Table A1

Items used in the analysis

Item Text	Scale
Neuroticism (X)	
I easily become depressed or discouraged.	1 (absolutely incorrect) to 5 (absolutely correct)
I am relaxed and don't let myself be worried by stress. (recoded)	
I worry a lot.	
Fear of love withdrawal (M)	
I'm often afraid my partner thinks I'm silly or stupid if I make a mistake.	1 (not at all) to 5 (absolutely)
Sometimes I'm afraid that my partner would rather spend time with others than with me.	
When I disappoint or annoy my partner, I am afraid that he/she won't like me anymore.	
Partnership autonomy (Y)	
My partner finds it quite all right if I stand up for my own interests in our partnership.	1 (not at all) to 5 (absolutely)
In our partnership I can usually do what I want.	
In our partnership, I can follow my own interests without my partner getting upset.	

Note. Retrieved from the pairfam scales manual.

Appendix B

Tables

Table B1

Population Values in the Simulation Study

	Effect Size				
Parameter	Small	Medium	Large	Prior (MNLFA)	Prior (MNLFA; diffuse)
$\gamma_0^{\alpha_i}$	2	2	2	$\mathcal{N}(2,2)$	$\mathcal{N}(0,5)$
$\gamma_1^{\alpha_i}$	.2	.5	.8	$\mathcal{N}(0,1)$	$\mathcal{N}(0,5)$
σ^{α_i}	.3	.3	.3	$\mathcal{HC}(0,1)$	$\mathcal{HC}(0,5)$
$\gamma_0^{\lambda_i}$	1	1	1	$\mathcal{N}(0,1)$	$\mathcal{N}(0,5)$
$\gamma_1^{\lambda_i}$	±.1	±.2	±.3	$\mathcal{N}(0,1)$	$\mathcal{N}(0,5)$
σ^{λ_i}	.3	.3	.3	$\mathcal{HC}(0,1)$	$\mathcal{HC}(0,5)$
$\gamma_0^{\sigma_{\varepsilon_i}}$	.4	.4	.4	$\mathcal{N}(0,1)$	$\mathcal{N}(0,5)$
$\gamma_1^{\sigma_{\varepsilon_i}}$	±.1	±.2	±.3	$\mathcal{N}(0,1)$	$\mathcal{N}(0,5)$
$\sigma^{\sigma_{\varepsilon_i}}$	.3	.3	.3	$\mathcal{HC}(0,1)$	$\mathcal{HC}(0,5)$
γ_0^{β}	.3	.3	.3	$\mathcal{N}(0,1)$	$\mathcal{N}(0,5)$
γ_1^{β}	±.1	±.2	±.3	$\mathcal{N}(0,1)$	$\mathcal{N}(0,5)$
σ^{β}	.3	.3	.3	$\mathcal{HC}(0,1)$	$\mathcal{HC}(0,5)$
γ_0^{σ}	.5	.5	.5	$\mathcal{N}(0,1)$	$\mathcal{N}(0,5)$
γ_1^{σ}	±.1	±.2	±.3	$\mathcal{N}(0,1)$	$\mathcal{N}(0,5)$
σ^{σ}	.3	.3	.3	$\mathcal{HC}(0,1)$	$\mathcal{HC}(0,5)$

Note. γ_0 = intercepts of moderation regressions; γ_1 = slopes of moderation regressions; σ = residual variation in moderation regressions. $\mathcal{N}(\mu, \sigma)$ = normal distribution prior with mean μ and standard deviation σ; $\mathcal{HC}(\mu, \sigma)$ = half-cauchy distribution prior with mean μ and standard deviation σ. For parameters with ± indicated, directions of effects of the moderator Z varied across latent variables.
